# Effectiveness of low‐density high‐throughput marker platform and easy‐to‐measure traits for genomic prediction of biomass yield in oat (*Avena sativa* L.)

**DOI:** 10.1002/tpg2.70179

**Published:** 2026-01-16

**Authors:** Samuel A. Adewale, Md Ali Babar, Diego Jarquin, Naeem Khan, Janam Prabhat Acharya, Sudip Kunwar, Jordan McBreen, Esteban Rios, Stephen Harrison, Noah DeWitt, Amir Ibrahim, Paul Murphy, Rick Boyles, Jason D. Fiedler, Raja Sekhar Nandety

**Affiliations:** ^1^ Plant Breeding Graduate Program University of Florida Gainesville Florida USA; ^2^ Department of Agronomy University of Florida Gainesville Florida USA; ^3^ School of Plant, Environmental and Soil Sciences Louisiana State University Baton Rouge Louisiana USA; ^4^ AgriLife Research Texas A&M University College Station Texas USA; ^5^ Department of Crop and Soil Sciences North Carolina State University Raleigh North Carolina USA; ^6^ Pee Dee Research and Education Center Clemson University Florence South Carolina USA; ^7^ USDA‐ARS Cereal Crops Improvement Research Unit, Edward T. Schafer Agricultural Research Center Fargo North Dakota USA

## Abstract

Genomic selection (GS) is a promising strategy for accelerating genetic gains of complex traits in breeding programs. Despite the recent advancements in high‐throughput genotyping technologies, the selection of the type of marker systems needed for GS remains challenging in breeding programs. In this study, we explored 3K array single nucleotide polymorphisms (SNPs) and genotyping by sequencing (GBS) SNP markers for genomic prediction of oat biomass yield using different statistical and machine learning approaches. An oat panel consisting of 420 lines was phenotyped for biomass‐related traits for 3 years and genotyped using two different marker platforms (3K array and GBS). Our results showed similar performance of both the 3K array and GBS‐based SNPs in terms of training population optimization, forward prediction, and univariate and multivariate genomic prediction of forage yield. The genomic best linear unbiased prediction (GBLUP), Bayes‐B, and random forest models gave similar predictive ability for dry matter yield (DMY) in different harvest–year combinations and for both marker platforms. The multivariate models involving various combinations of secondary traits (simple breeders' field notes and data) resulted in more than twofold increases in predictive abilities compared to the univariate models. Comparison of the 25% top‐performing observed and predicted genotypes showed a higher overlap percentage (30.10%–66.99%) for multivariate GBLUP models compared to the univariate models (27.18%–51.46%). This further elucidates the great potential of multivariate GS models incorporating the more robust and easily reproducible 3K array SNP markers for improving the genetic gains of DMY in breeding programs.

## Core Ideas

- Illumina 3K single nucleotide sequencing (SNP) array and genotyping by sequencing (GBS) SNP markers performed similarly for genetic diversity, as well as training population optimization.
- Illumina 3K and GBS SNPs demonstrated comparable predictive abilities for dry matter yield (DMY) using univariate and multivariate models.
- Multivariate prediction models had higher predictive ability for oat DMY than univariate models.
- The reproducibility, speed, and ease of production, as well as the lower marker density of the 3K array, would enhance its utilization for genomic selection (GS).

## Plain Language Summary

In the Southern United States, oats have been extensively used as a forage crop for animal feed. Nevertheless, collecting phenotypic data for aboveground biomass improvement in oat breeding programs is destructive, labor‐intensive, and takes a lengthy time. We investigated two different marker genotyping platforms for genomic prediction of oat biomass yield using different univariate and multivariate models. The inclusion of easy‐to‐measure secondary traits in our models resulted in a better ability to determine oat genotypes that will give higher biomass yield. Comparable predictive abilities were observed for both marker genotyping methods. Thus, the more robust 3K array genotyping method with lower marker density could be utilized by breeders for selection of oat genotypes in early generations using secondary traits in genomic selection to reduce cost, shorten the time required to develop new varieties, and eventually boost the availability of nutritious oat forage for the livestock industry.

**Abbreviations**
BLUEs: best linear unbiased estimates
DMY: dry matter yield
GBLUP: genomic best linear unbiased prediction
GBS: genotyping by sequencing
GC: ground cover
GH: growth habit
GS: genomic selection
LD: linkage disequilibrium
PH: plant height
QTL: quantitative trait loci
RF: random forest
SNP: single nucleotide polymorphism
SOAP: Southern Oat Association Panel
XGBoost: extreme gradient boosting

## 1 INTRODUCTION

Oat (*Avena sativa* L.) is the sixth most important cereal crop in the world, due to its highly nutritious grains for humans and forage for animal feed (J. Peng et al., 2024). In the southern United States, especially in areas stretching from Texas to Florida and up through the Carolinas, oats are primarily used as a forage crop for grazing, silage, and hay for beef, dairy, and other classes of livestock. Livestock forage production is one of the largest contributors to the agricultural economy in the southern United States. Due to the dormancy of warm‐season perennial grasses, producers often face a lack of high‐quality, protein‐rich forage supply during late fall to mid‐spring (mid‐November to April). Oats provide excellent forage from November to mid‐April, which is especially important at a time when other high‐protein feeds such as bermudagrass and bahiagrass are scarce in the southern United States (Dubeux et al., 2016). Compared to other small‐grain winter cereals, oats are preferred for livestock grazing due to their rapid growth, high tillering capacity, and excellent recovery ability after grazing (Oral, 2024). Oat forage has several beneficial characteristics, such as high yield, elevated levels of crude protein, abundant water‐soluble carbohydrates, and a substantial amount of digestible neutral dietary fiber, making it a superior feed option due to its high palatability (Sood et al., 2022). In addition, its reasonably short growth period and better adaptation to abiotic stresses have contributed to its widespread recognition as a valuable forage crop (Oral, 2024; Zhang et al., 2024).

Forage yield is a crucial and difficult trait to measure and improve, requiring multiple cuts from individual plots from different trials over the growing seasons and years (Kim et al., 2014). In multi‐cut forage trials, oat biomass is harvested two to four times during the growing season to determine fall, winter, as well as early and late spring forage yield (Babar et al., 2023a, 2023b; Babar et al., 2024). The advent of low‐cost and high‐throughput genotyping technologies has revolutionized breeding programs for obtaining a considerable amount of genomic data to aid selection decisions and approaches such as genomic selection (GS) (Crossa et al., 2025; Rio et al., 2021). Plant breeders can thus improve selection accuracy and intensity and reduce the generation interval for complex traits enhancement, thus increasing breeding efficiency for improving food production in the future (Sørensen et al., 2023). Compared to other important cereal crops such as rice, maize, and wheat, the breeding and genomics of cultivated oats are falling behind, predominantly due to their sizable, repeat‐rich, and complex genome, as well as low research funding (Hu et al., 2020; Li et al., 2025; J. Peng et al., 2024; Wang et al., 2023). Similarly, the existence of nonhomologous pairing due to the high level of closeness between the A and D genomes leads to significant rearrangements in the hexaploid oat chromosomes compared to the basic diploid ancestral chromosomes (Leggett & Markhand, 1995; Sowa & Paczos‐Grzęda, 2020). However, recent advances in molecular marker sequencing technology have resulted in the generation of some high‐quality and fully annotated oat genomes, such as the Sanfensan oat genome and pangenome (Avni et al., 2025; Y. Peng et al., 2022), leading to improved genome‐wide marker detection for various complex traits in oats (Yan et al., 2023).

Various genotyping technologies have been utilized routinely for whole‐genome characterization. These include parallel assays that target semi‐random polymorphisms, such as amplified fragment length polymorphism and diversity array technology, as well as parallel assays for specific single nucleotide polymorphism (SNP) (Huang et al., 2014). These different genotyping platforms show technical merits and demerits. Those that identify semi‐random polymorphisms may not give a sufficient number of markers throughout the genome, and the technology may not be transferable. The SNP assay developed through genotyping by sequencing (GBS) platform has been used for genomic prediction of several traits in oats (Brzozowski et al., 2023; Haikka et al., 2020; Huang et al., 2014; Mellers et al., 2020; Rio et al., 2021). Recently, a genome‐wide Illumina Infinium XT genotyping array that permits the combination of custom multispecies variants was designed by the United States Department of Agriculture–Agricultural Research Service (USDA‐ARS) North Central Small Grains Genotyping Laboratory, Fargo, ND (Bazzer et al., 2025). This SoyWheOatBar 3K array consists of evenly spaced and informative SNPs for soybean, wheat, oat, and barley, with approximately 3000 SNPs built in the array for each crop (J. Fiedler, personal communication, 2024).

GS is an important marker‐based breeding tool that utilizes whole‐genome DNA markers and phenotypic information of a training population to predict the performance of a testing breeding population (with only genomic information) using statistical models (Meuwissen et al., 2001; Sørensen et al., 2023). It is a promising strategy for accelerating the genetic improvement of biomass yield compared to marker‐assisted or phenotypic selection (Asoro et al., 2013; Filho et al., 2023; Maulana et al., 2019; Murad Leite Andrade et al., 2022). GS has the potential to substantially increase the rate of genetic gain for quantitative traits, especially those traits that are costly and challenging to phenotype and cannot be evaluated at earlier stages of the breeding program (Gill et al., 2023). Therefore, integrating GS into the oat breeding program has the potential to lead to the development of superior varieties with better agronomic characteristics and quality in a shorter time (Rocha et al., 2023).

**Core Ideas**

- Illumina 3K single nucleotide sequencing (SNP) array and genotyping by sequencing (GBS) SNP markers performed similarly for genetic diversity, as well as training population optimization.
- Illumina 3K and GBS SNPs demonstrated comparable predictive abilities for dry matter yield (DMY) using univariate and multivariate models.
- Multivariate prediction models had higher predictive ability for oat DMY than univariate models.
- The reproducibility, speed, and ease of production, as well as the lower marker density of the 3K array, would enhance its utilization for genomic selection (GS).

Genomic prediction is generally conducted using univariate models such as ridge‐regression best linear unbiased prediction or its computationally convenient parameterization known as genomic best linear unbiased prediction (GBLUP), other Bayesian models, and random forest (RF) for predicting single traits (Atanda et al., 2022; Malauna et al., 2019). Nevertheless, in breeding programs, multiple traits that are genetically correlated are measured. Previous studies have evaluated the use of secondary traits as independent covariate predictors along with marker data in genomic prediction models to improve the prediction accuracy of target traits of interest (Guo et al., 2020; Lozada & Carter, 2019; McBreen et al., 2025a, 2025b; Sandhu et al., 2021; Zhang‐Biehn et al., 2021). Incorporating easy‐to‐measure secondary traits in genomic prediction can be crucial for improving the prediction accuracy of a correlated primary trait, especially if the primary trait is difficult or expensive to measure and/or has low heritability (Fernandes et al., 2018). Plant height (PH) is usually highly associated with biomass yield, and it has been used in previous studies as a covariate for biomass yield prediction in addition to genomic data in the validation population (Fernandes et al., 2018; Galán et al., 2020). Visual rating of canopy ground cover (GC) and growth habit (GH) are other easy‐to‐measure breeders’ traits that could be used in genomic prediction models for improving dry matter yield (DMY). Utilizing information from correlated secondary traits in GS has resulted in improved prediction accuracy varying from 24% to 59% compared to univariate models for DMY (Arojju et al., 2020; Galán et al., 2020). Lozada and Carter (2019) obtained a 50% increase in prediction accuracy using secondary traits as independent predictors in GS models compared to the typical univariate models. To the best of our knowledge, there is no report on the implementation of multivariate genomic prediction for DMY improvement in oats.

With the recent advancements in high‐throughput genotyping technologies, selecting the most appropriate marker platform for implementing GS is crucial in breeding programs. Bazzer et al. (2025) compared the USDA‐ARS 3K array and GBS marker platforms for genomic prediction and reported that the reduced number of markers in the 3K array compared to GBS did not decrease the prediction accuracy of β‐glucan content in oat. Thus, the 3K array platform is a promising genotyping platform for implementing GS in oat breeding programs due to its high reproducibility, speed, and ease of production, as well as the lower marker calls every year. However, the choice of the marker platform would depend on the goal of the breeders, cost, data computation resources, and germplasm pool of the breeding program. The objectives of this study were to (i) compare the 3K array and GBS marker platforms for population structure patterns and assess the effects of marker density as well as varying number of individuals on genomic predictive abilities of DMY using both marker platforms, (ii) determine the genomic predictive abilities of DMY using different statistical and machine learning models with both marker platforms, and (iii) integrate easy‐to‐measure secondary traits as predictors in different GS models and compare with univariate GS models.

## 2 MATERIALS AND METHODS

### 2.1 Plant materials and phenotyping

The present study used a well‐characterized Southern Oat Association Panel (SOAP) consisting of 420 genotypes mostly developed by the University of Florida, Louisiana State University, Texas A&M University, North Carolina State University, and Clemson University, and adapted to the southern United States (Table S1). This germplasm panel contains mostly elite lines and commercial varieties with favorable agronomic performance. In addition to the elite lines from those five public breeding programs, several commercial varieties adapted to different oat‐growing areas in the United States were also included in the panel. The panel was evaluated for 3 years (2022–2025) between October and March of each growing season at the Plant Science Research and Education Unit, Citra, Florida, using an alpha lattice design with two replications. Data were collected on GH, PH, GC, and DMY. PH (cm) was measured as the average distance from the ground level to the tip of three randomly selected plants in a plot before biomass harvesting. GH was scored visually on a scale of 1–9, 1 = *erect/spring type*, 5 = *semi‐prostrate/facultative type*, and 9 = *prostrate/winter type*. The GH data were collected only before the first biomass harvest, as GH was not expected to change over time. The GC was scored before biomass harvesting on a scale of 1–9, 1 = *10% of the plot area covered by the oat plants* and 9 = *90%–100% of the plot area covered by the oat plants*. Biomass was harvested very early in January (first harvest, H1), mid‐February (second harvest, H2), and late March (third harvest, H3), as these times represent the late fall growth and early grazing, regrowth, and rapid spring growth periods. The GC and PH for the first biomass harvest were not collected in the first year of the experiment. The biomass was harvested from an area of 3.25 m^2^ using a forage harvester. At each harvest period, total fresh weight per plot was recorded, and about 500 g of fresh subsamples were collected immediately. The subsamples were dried at about 55°C until constant weight was achieved, approximately 1 week. Dry matter content was calculated and used to determine DMY (kg/ha) per plot.

### 2.2 Genotyping

The seeds of each line in the SOAP panel were sent to the USDA‐ARS Small Grains Genotyping Laboratory at Fargo, ND, for genotyping. Genomic DNA of each inbred line was extracted from bulked leaf samples of 2‐week old seedlings using a modified sodium dodecyl sulfate method (Bazzer et al., 2025). DNA extraction and library preparation for the double digest (PstI‐MspI) GBS protocol were followed as previously described by Huang et al. (2014). The quantity and quality of each DNA sample were assessed using PicoGreen assay and nanophotometer (NanoDrop 2000C, Thermo Scientific) (Wang et al., 2023). The panel of breeding lines was genotyped using two different genotyping platforms to deliver genome‐wide SNP markers.

The lines were first genotyped using the GBS platform, which is based on genome complexity reduction with restriction enzymes PstI and MspI (Elshire et al., 2011). The 96‐plex libraries were prepared as described by Poland et al. (2012). The lines were also genotyped utilizing the USDASoyWheOatBar‐3 K Illumina iSelect array (3K oat SNP array) designed by the USDA‐ARS Small Grains Genotyping Laboratory, Fargo, ND (J. Fiedler, personal communication, 2024). For SNP calling, paired‐end sequencing of the GBS libraries was performed on an Illumina HiSeq 2000 system with 150 bp paired ends. The sequencing reads were aligned to the *A. sativa* PepsiCo OT3098 v2 genome using bowtie2. Raw single‐nucleotide variants and indels were called using SAMtools and bcftools (Bazzer et al., 2025). SNPs were visualized using TASSEL v5.0 (trait analysis by association, evolution, and linkage) software. Only 409 lines out of the 420 lines phenotyped for biomass‐related traits had SNP marker data after genotyping. The raw GBS genotypic data consisted of 51,533 SNP markers, and the 3K oat SNP array consisted of 2989 SNP markers. SNP markers with greater than 10% heterozygosity, greater than 30% missing data, and minor allele frequency of less than 5% were eliminated. Unmapped and monomorphic markers were also removed. The resulting 12,415 and 1801 SNPs from the GBS and 3K array platforms, respectively, were imputed using LD‐kNNi (Linkage Disequilibrium k‐Nearest Neighbors imputation) approach in TASSEL 5.0 software (Bradbury et al., 2007). Before imputation, the proportion of missing marker data in the GBS and 3K SNP marker data was 11.9% and 1.4%, respectively.

### 2.3 Phenotypic data analysis

Best linear unbiased estimates (BLUEs) for DMY (kg/ha) and other related traits from each harvest in each year were obtained using the lmer function in the lme4 R package (Bates et al., 2015).

The statistical model used is specified as follows:

(1) $$Y_{ijh}=\mu +\gamma_{j}+b_{jh}+\tau_{i}+e_{ijh}$$

where $Y_{ijh}$ represents the observed performance of the *i*th genotype in the *h*th block nested within the *j*th replicate, μ is the overall mean, $\gamma_{j}$ corresponds to the effect of the *j*th replicate, $b_{jh}$ is the effect of *h*th block nested within *j*th replicate, $\tau_{i}$ is the fixed effect of the *i*th genotype, and $e_{ijh}$ captured the unexplained variability or residual plot error. The random effects of the blocks and the residual error are represented as $b_{jh}∼N\left(0,\sigma_{b}^{2}\right)\;\mathrm{and}\;e_{ijh}∼N\left(0,\sigma^{2}\right)$, respectively.

Broad‐sense heritability of the traits was estimated based on variance components obtained using the previous linear predictor with the genotypic effect treated as random, $\tau_{i}∼N\left(0,\sigma_{g}^{2}\right)$. In addition, environments (harvest × year) and genotype × environment interaction effects were treated as random for estimating broad‐sense heritability across all environments. The broad‐sense heritability was calculated for each harvest and across all environments as described by Hallauer et al. (2010) as follows:

(2) $$H^{2}=\frac{\sigma_{g}^{2}}{\sigma_{g}^{2}+\frac{\sigma_{ge}^{2}}{e}+\frac{\sigma^{2}}{er}}$$

where $\sigma_{g}^{2}$ is the genotypic variance, $\sigma^{2}$ is the error variance, $\sigma_{ge}^{2}$ is the variance due to genotype × environment interaction effects, e is the number of environments, and *r* is the number of replications.

### 2.4 Linkage disequilibrium and population structure analyses

Pairwise linkage disequilibrium (LD) across the genome was estimated utilizing squared allele frequency correlation (*r* ^2^) between pairs of SNPs in TASSEL 5.2.93 software (Bradbury et al., 2007) using a sliding window of 50 markers. The *r* ^2^ values were plotted against the genetic distance to determine the LD decay, and the LOESS curve was fitted using R software (R Core Team, 2024). LD decay was identified as the physical genomic distance at which the *r* ^2^ decreased to half of its maximum value, where *r* ^2^ = 1, indicating complete LD, and *r* ^2^ = 0, indicating absence of LD (Nouraei et al., 2024). Principal component analysis (PCA) was carried out using the ggfortify R package (Tang et al., 2016), and the percentage of genotypic variation explained by the first two principal components (PCs) was obtained to enable comparison between the two genotyping marker platforms used in this study. The PCA analysis was also conducted using *K*‐means clustering to validate the number of clusters utilizing the factoextra R package (Kassambara & Mundt, 2020). The optimal number of clusters was determined using the elbow method. In addition, the Mantel test was used to compare the genomic relationship matrices derived from the 3K array and GBS SNP marker data (Mantel, 1967).

### 2.5 Training population and marker density optimization

To understand the effect of varying marker density and training population sizes on the predictive ability of DMY using 3K and GBS genotyping platforms, various marker densities were implemented by selecting at random a subset of markers to produce a genomic matrix. Varying marker sizes selected for the GBS SNPs include 20, 100, 200, 500, 800, 1200, 1801, 3000, 5000, 8000, and 12,415. For the 3K SNP array, SNP marker sizes selected include 20, 100, 200, 500, 800, 1200, and 1801. For both marker platforms, a cumulative method was used to prevent bias resulting from the informative nature of the markers, such that the first subset of markers randomly selected was included in the next subset and so on (Sipowicz et al., 2025). The oat panel was randomly subdivided into a training set (80% of observations) and a validation set (20% of observations) for each marker subset. Random sampling of the marker sets was repeated 20 times for each scenario. Similarly, the effect of training population size on the predictive ability of the GS model was assessed using seven varying population subsets, which include 50, 100, 150, 200, 250, 300, and 350 as the training set. The remaining individuals in the dataset after selecting each training subset were used as the validation sets (Adunola et al., 2024). Each training population subset was randomly selected to predict the validation set using the GBLUP model. This was also repeated 20 times. To ensure proper genome coverage and that the overall population structure was captured, the complete set of markers from both genotyping platforms and all genotypes in the oat panel were used for subsequent genomic prediction analyses.

### 2.6 Genomic prediction using the complete set of markers

#### 2.6.1 Single‐trait genomic prediction

Both statistical and machine learning GS models were implemented using BLUEs obtained for the traits and genome‐wide marker information to assess the levels of predictive ability that can be accomplished. Four single‐trait genomic prediction models, including GBLUP, Bayes‐B, RF, and extreme gradient boosting (XGBoost), were used in this study. These models were compared because of their different assumptions in predicting genotypic performance. The robust and computationally efficient GBLUP model, which assumes that all markers have an effect on trait variability and that these effects follow a normal distribution with a common variance was used as the baseline model (Verbriggheet al., 2025). The GBLUP and Bayes B models were performed using the BGLR R‐package (Pérez & de los Campos, 2014; Rio et al., 2021). The model equation for GBLUP is expressed as follows:

(3) y=μ1+Zg+e

where y is the vector of BLUEs for a single trait, μ is the grand mean, **1** is a vector of ones, and **e** is the vector of residual errors with $e∼N(0,\sigma_{e}^{2})$. **Z** is the incidence matrix for genotype effects (linking phenotypes to breeding values), **g** is the vector of genomic effects $g_{i}$ such that $g=\left\{g_{i}\right\}∼N\left(0,\;G\sigma_{g}^{2}\right)$ , where **G** is the genomic relationship matrix (VanRaden, 2008) and $\sigma_{g}^{2}$ is the additive genetic variance. The model equation for Bayes B is expressed as follows:

(4) $$y=\mu 1+X_{\beta }+e$$

***y*,** μ and e are the same as in Equation (3). X is the genotypic matrix corresponding to $\beta ,\;\beta =\left\{\beta_{i}\right\}∼N\left(0,\sigma_{\beta i}^{2}\right)$ is the vector of fixed effects and $\sigma_{\beta i}^{2}$ is the variance of the *i*th marker effect. In Bayes‐B, only a small fraction of the markers (1 − π) can have a nonzero effect on the trait variability with a variance specific to the individual marker. Thus, potentially accounting for some markers with large effects (Rio et al., 2021). The Bayes B model was implemented with 2000 burn‐ins and 12,000 MCMC (Marvok chain Monte Carlo) iterations of the Gibbs sampler.

The RF, a tree‐based machine learning model, was performed using the “randomForest” R package (Liaw & Wiener, 2002). The RF model equation is expressed as follows:

(5) $${\hat{y}}_{i}=\frac{1}{M}\;\sum_{m=1}^{M}t_{m}\left(x_{i}\right)$$

where${\hat{\;y}}_{i}$ is the predicted value of RF regression with input $x_{i}$, $t_{m}\left(x_{i}\right)$ represents each regression tree, and *M* denotes the number of decision trees in the forest (Montesinos‐López et al., 2022). The RF model uses an ensemble of decision trees that are individually trained utilizing bootstrapping (random sampling with replacement of samples) and a random subset of SNPs. The clustering property of trees allows the RF model to sufficiently capture correlations and interactions among predictors. The test sample predictions are averaged to produce a final estimate to reduce the possibility of overfitting. A total of 500 trees were used in this study.

The XGBoost model was implemented using “XGBoost” package in R. The XGBoost model is based on the gradient boosting decision trees but performs a more powerful prediction by incorporating a substantial number of weak learners (that is, decision trees). The XGBoost model equation is expressed as follows:

(6) $${\hat{y}}_{i}=\sum_{k=1}^{K}f_{k}\left(x_{i}\right)$$

where ${\hat{y}}_{i}$ denotes the predicted value of the XGBoost model, $f_{k}\left(x_{i}\right)$ represents the base model for the decision tree representation, and K indicates the number of decision trees (B. Liu et al., 2025; Zhou et al., 2023). This ensemble method uses regression trees by varying the residual of an initial model. The XGBoost is a more powerful prediction model than the gradient boosting decision tree. It integrates a large number of decision trees, and each new decision tree is trained based on the residual of the prediction from the previous trees, and the response variable involves a combination of a substantial number of trees. The prediction error of the model is therefore further minimized in each iteration. The number of trees sampled was 500, with a learning rate of 0.1 and a depth of 6.

#### 2.6.2 Multivariate genomic prediction

In the multivariate GS model, the secondary traits (PH, GH, and GC) were fitted as fixed effects covariate predictors to predict the primary trait (DMY) as follows:

(7) y=μ+Wγ+Zg+e.

Here, all the terms are the same as in Equation (3), with the inclusion of ***W***, which represents the design matrix associating the fixed effects of the secondary traits and ***γ*** representing the vector of fixed‐effect coefficients of the secondary traits. Both the SNP marker data and the secondary traits (individual traits and in different combinations) were used as independent predictors in the model to obtain genomic estimated breeding values for the genotypes (Lozada & Carter, 2019; Zhang‐Biehn et al., 2021). The multivariate model was performed using the BGLR library in R with 12,000 MCMC iterations and 2000 burn‐in iterations (Pérez & de los Campos et al., 2014). To compare the performance of the linear model with the machine learning model, the multiple trait prediction was also performed using the RF model with the genomic information and secondary traits included as independent covariate predictors using the “randomForest” R package (Liaw & Wiener, 2002). We also compared the ability of the univariate and multivariate GBLUP, as well as RF prediction models, in predicting oat lines with the highest DMY. The best 25% of the lines were ranked and selected based on observed and predicted DMY from each harvest in individual years to determine the similarity percentage between the observed and predicted genotypes.

#### 2.6.3 Cross‐validation scheme and forward prediction

Predictive abilities of the models were estimated from the correlation between genomic‐estimated breeding values and the actual phenotypic values of individuals in a validation set using a cross‐validation strategy. For the single‐trait models (GBLUP, Bayes‐B, RF, and XGBoost), a fivefold cross‐validation approach was implemented in which 80% of the oat genotypes were used as the training set and the remaining 20% as the test set. This procedure was repeated 10 times. For the multivariate models cross‐validation scenario, the training set had phenotypic information of the primary trait and the secondary traits, as well as the genotypic information. The validation set had only phenotypic information of the secondary traits and genotypic data. The oat panel was randomly subdivided into a training set (80% of observations) and a validation set (20% of observations), and the process was repeated 100 times to obtain the predictive ability of each model for different combinations of the three secondary traits (PH, GH, and GC). For the univariate models (GBLUP and RF), the same cross‐validation approach as the multivariate models was used, except for the inclusion of secondary trait predictors. To determine the ability of the GBLUP model to predict DMY in future harvests and years, a forward prediction strategy that employs data from the earlier harvest(s) or years to predict subsequent harvests/years' performance was implemented (McBreen et al., 2025a). These independent predictions were performed by using DMY data from harvests 1 and 2 individually and in combination to predict harvest 3 DMY in each year. Similarly, this approach was applied to predict DMY in the year 2025 using DMY data from the years 2023 and 2024 individually and altogether.

## 3 RESULTS

### 3.1 Marker distribution, PCs, and LD analyses

Quality filtering of the markers resulted in 1801 3K SNP markers and 12,415 GBS SNP markers distributed among the 21 chromosomes for further analyses. For the 3K platform, chromosomes 6C and 5C had the highest marker numbers, 135 and 122, respectively, whereas chromosomes 5C and 4C had the highest marker numbers, 1216 and 1098, respectively, for the GBS platform (Table 1). The lowest marker number was on chromosome 6D for both marker platforms, with 44 markers for the 3K platform and 118 markers for the GBS platform. The overall linkage group length for both marker platforms was similar, with 10,596.56 and 10,585.19 Mb for the 3K and GBS SNP markers, respectively. For the 3K SNPs, chromosome 3D had the highest average marker distance per chromosome (9.43 Mb) while chromosome 4A had the lowest average marker distance per chromosome (4.38 Mb). The highest average marker distance per chromosome (2.53 Mb) was found on chromosome 6D for the GBS SNPs, while chromosome 5C had the lowest average marker distance per chromosome (0.50 Mb). Across the genome, the average marker distances were 5.95 and 0.85 Mb for the 3K and GBS SNP marker platforms, respectively. This indicated less genome coverage by the 3K SNP array.

**TABLE 1:** Marker distribution on different chromosomes based on 3K and genotyping by sequencing (GBS) marker platforms.

	3K			GBS		
Chromosome	Marker number	Linkage group length, Mb	Average marker distance, Mb	Marker number	Linkage group length, Mb	Average marker distance, Mb
1A	94	535.87	5.76	711	539.25	0.76
1C	77	457.65	6.02	792	456.41	0.58
1D	106	479.37	4.57	484	473.63	0.98
2A	48	440.97	9.38	240	438.65	1.84
2C	95	559.39	5.95	923	560.43	0.61
2D	91	529.69	5.89	555	528.60	0.95
3A	66	417.42	6.42	176	421.91	2.41
3C	100	635.13	6.42	892	637.54	0.72
3D	51	471.63	9.43	441	477.73	1.09
4A	106	459.56	4.38	378	384.49	1.02
4C	104	709.08	6.88	1098	709.70	0.65
4D	87	423.28	4.92	571	441.77	0.78
5A	72	478.71	6.74	305	479.62	1.58
5C	122	610.10	5.04	1216	610.31	0.50
5D	87	495.95	5.77	556	491.53	0.89
6A	71	427.03	6.10	293	443.56	1.52
6C	135	615.76	4.60	1096	622.38	0.57
6D	44	295.57	6.87	118	296.54	2.53
7A	68	490.58	7.32	345	492.72	1.43
7C	112	548.34	4.94	915	551.24	0.60
7D	65	515.50	8.05	310	527.16	1.71
Whole genome	1801	10,596.56	5.95	12,415	10,585.19	0.85

*Note*: 3K, 3K SNP array‐based marker platform.

**FIGURE 1:**
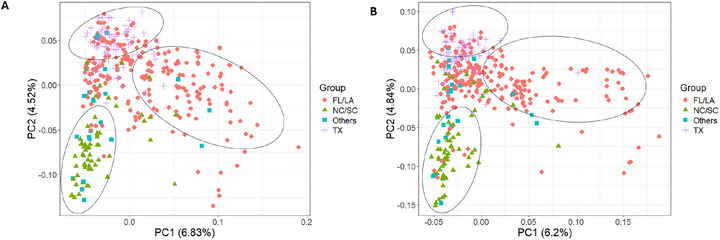
Scatter plots from the principal component (PC) analyses showing population structure in the Southern Oat Association Panel (SOAP) based on 1801 single nucleotide polymorphisms (SNPs) from the 3K marker platform (A) and 12,415 genotyping by sequencing (GBS)‐based SNP markers (B). The color‐coded points represent the origin of the lines: University of Florida (FL), Louisiana State University (LA), North Carolina State University (NC), Clemson University (SC), Texas A&M University (TX), and others represent historic lines from different breeding programs across the United States.

The PCAs using each of the marker platforms similarly grouped the lines into three subclusters (Figure 1). The first and largest subpopulation consisted of lines from the University of Florida and Louisiana State University (FL/LA) small grains breeding programs. Subpopulation 2 consisted of lines from Texas A&M University (TX), and the lines in Subpopulation 3 are mostly from North Carolina State University (NC) and Clemson University, South Carolina (SC) breeding programs. Optimal number of clusters (*K* = 3) in the oat panel using the two marker platforms was further validated by the elbow method and then displayed using *K*‐means clustering (Figures S1 and S2).

The first and second PCs explained 6.83% and 4.52% of the total genotypic variation, respectively, in the population using the 3K markers, whereas PC1 and PC2 explained 6.2% and 4.84% of the total genotypic variation, respectively, using the GBS SNP markers (Figure 1; Figure S2). Similarly, the results of the Mantel test showed a highly significant correlation (0.80**) between the genomic relationship matrices obtained from the two marker platforms (Table S2). The lack of a strong association among the lines in the oat panel revealed its suitability for assessing the performance of genomic prediction models.

**FIGURE 2:**
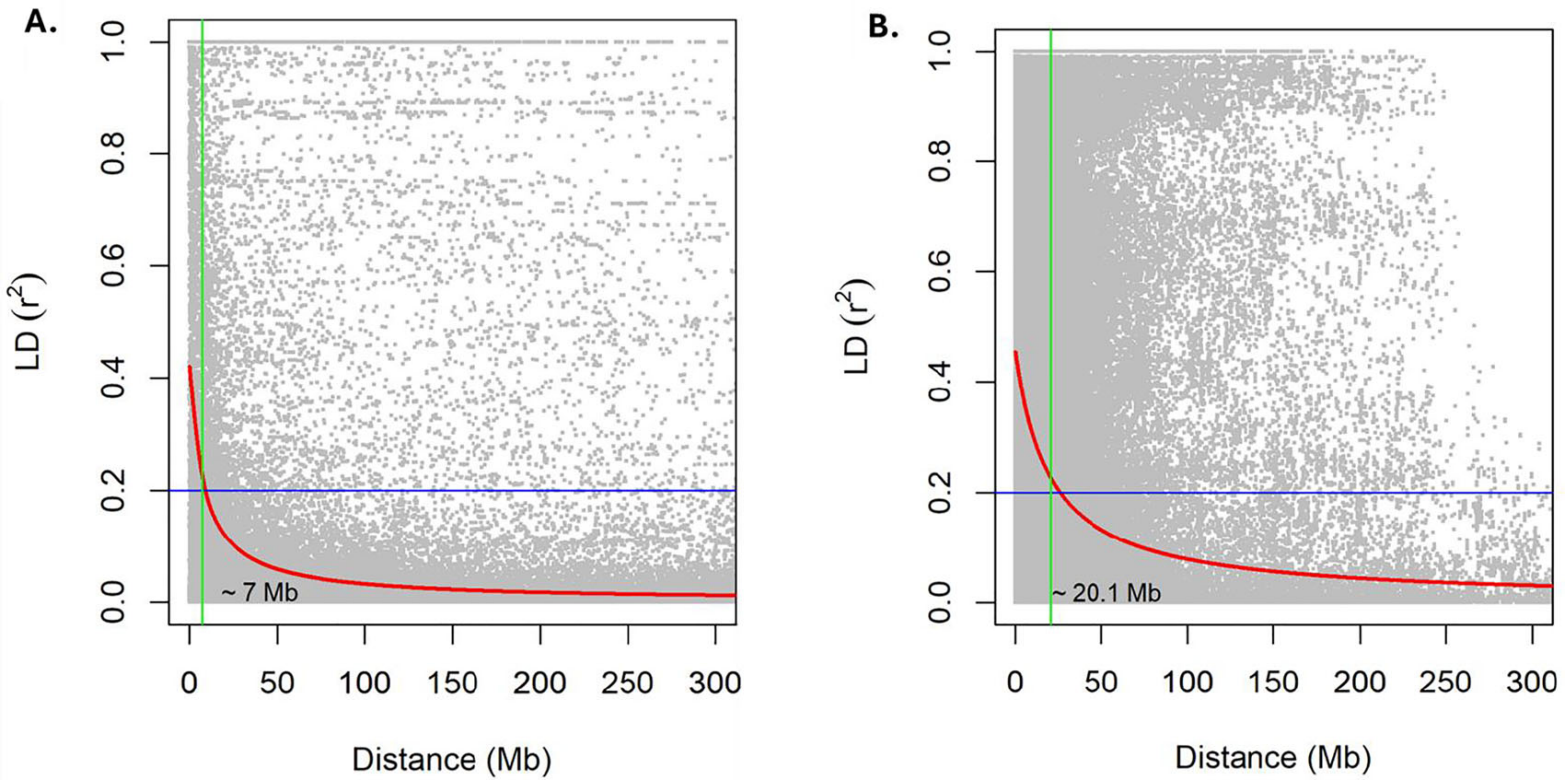
Linkage disequilibrium (LD) decay plot across the oat genome showing *r*‐squared between pairs of polymorphic sites plotted against physical distance (Mb) between the sites using 1801 single nucleotide polymorphisms (SNPs) from the 3K marker platform (A) and 12415 genotyping by sequencing (GBS)‐based SNP markers (B).

The 3K and GBS‐SNPs were used to assess the extent of LD in the SOAP. LD was calculated as the squared correlation coefficient (*r* ^2^) among pairs of markers. At approximately *r* ^2^ = 0.2, genome‐wide LD decay was observed at approximately 20.1 and 7.0 Mb for the GBS and the 3K array marker platforms, respectively (Figure 2).

### 3.2 Phenotypic performance of traits in the oat panel

The agronomic performance of the oat panel varied for various traits in different environments (Table S3; Figures S3–S5). The average PH varied from 20.98 cm in the second harvest (H2) of year 2023 to 89.20 cm in the last harvest (H3) of year 2023. In the year 2024, PH ranged from 16.84 cm in the first harvest (H1) to 92.05 cm in the third harvest (H3). In 2025, PH varied from 25.10 cm in harvest 2 (H2) to 80.72 cm in harvest 1 (H1). The GH scores varied from 2.00 to 8.50 in 2023, 2.55 to 8.02 in 2024, and 1.85 to 8.54 in 2025.

The GC scores ranged from 1.87 for harvest 3 (H3) to 8.65 for harvest 1 (H1) in 2024 and from 2.51 for harvest 3 (H3) to 9.16 for harvest 1 (H1) in the year 2025. The GC data were not collected in 2023. The DMY varied from 143.58 kg/ha for harvest 1 (H1) to 5779.53 kg/ha for harvest 2 (H2) in 2023, from 302.43 kg/ha for harvest 1 (H1) to 5515.38 kg/ha for harvest 3 (H3) in 2024, and from 407.40 kg/ha for harvest 1 (H1) to 4228.92 kg/ha for harvest 3 (H3) in 2025 (Table S3). Broad‐sense heritability was estimated for DMY, GC, PH, and GH for each harvest period in each year and across all environments (Table S4). The highest heritability estimates observed for DMY were 0.39 in 2023, 0.47 in 2024, and 0.32 in 2025. The heritability of DMY was lower compared to other secondary traits. For PH, the highest heritability estimates obtained were 0.49 for harvest 3 (H3) in 2023, 0.77 for harvest 2 (H2) in 2024, and 0.71 for harvest 1 (H1) in 2025. The GH showed the highest heritability estimates among the traits evaluated, ranging from 0.73 in 2025 to 0.81 in 2024. The highest heritability estimates of 0.60 for harvest 3 (H3) and 0.58 for harvest 2 (H2) were observed in 2024 and 2025, respectively, for GC. Across all environments, heritability estimate of 0.16 was observed for biomass yield, 0.43 for PH, 0.63 for GC, and 0.55 for GH. Pearson correlation showed a moderate to high positive relationship between DMY and PH (0.38**–0.72**) and low to high positive associations with GC (0.01–0.73**) in the different harvest periods and years (Figures S6–S8). Moderate to high negative correlations were observed only between GH and DMY (−0.32** to −0.65**) obtained from the first harvest of each year.

### 3.3 Marker and training subset optimization

**FIGURE 3:**
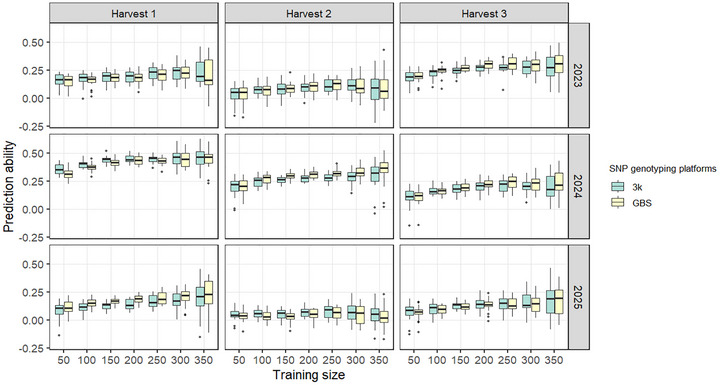
Predictive abilities of oat dry matter yield using varying population sizes for three different harvests, each in three years. 3K, 3K single nucleotide polymorphism (SNP) array‐based marker platform with 1801 SNPs; GBS, genotyping by sequencing SNP marker platform with 12,415 SNPs.

**FIGURE 4:**
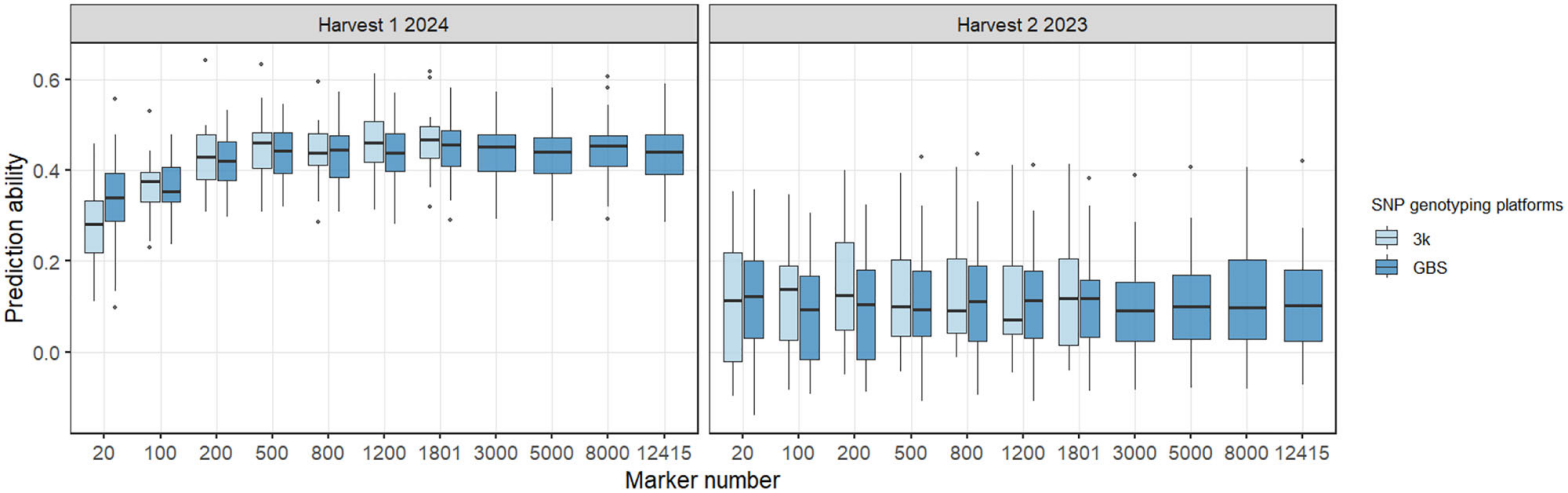
Effect of varying marker numbers on predictive abilities of dry matter yield based on genotyping by sequencing (GBS) and 3K marker platforms. Single nucleotide polymorphism (SNP) density effect on predictive ability using 2024‐harvest 1 (H1), environment with the highest predictive ability, and 2023‐harvest 2, environment with the lowest predictive ability for dry matter yield; 3K, 3K SNP array‐based marker platform.

The predictive ability of DMY with varying marker numbers and training population sizes was examined using the GBLUP model. An increase in training population sizes resulted in increased predictive abilities in most scenarios (different harvest periods and marker platforms) except for the 2025 harvest 2 scenario, which showed some inconsistencies in the predictive abilities pattern observed (Figure 3; Table S5). The training population size of 50 had the lowest predictive ability in most scenarios, reaching a peak between population sizes of 250–350. The effect of marker density on predictive ability was assessed using 2024 harvest 1 (H1) data, which gave the highest predictive ability, and 2023 harvest 2 (H2) with the lowest predictive ability (Figure 4; Table S6). For the 2024 harvest 2, an increase in predictive ability was generally observed as marker number increases for both marker platforms up to marker number 1801, after which slight decreases in predictive ability began to set in for the GBS markers. The 20 randomly selected markers had the lowest predictive ability of 0.276 and 0.335 for the 3K and GBS platforms, respectively, whereas marker number 1801 gave the highest predictive ability of 0.466 for the 3K markers and 0.448 for the GBS SNPs. For the 2023 harvest 2, with the lowest overall predictive ability, there was no significant increase in predictive abilities with an increase in the marker numbers for both platforms. To ensure proper genome coverage and capture the overall population structure, the complete set of markers from both genotyping platforms and all genotypes in the oat panel were used for subsequent genomic prediction analyses.

### 3.4 Predictive abilities of DMY using different statistical and machine learning models

**FIGURE 5:**
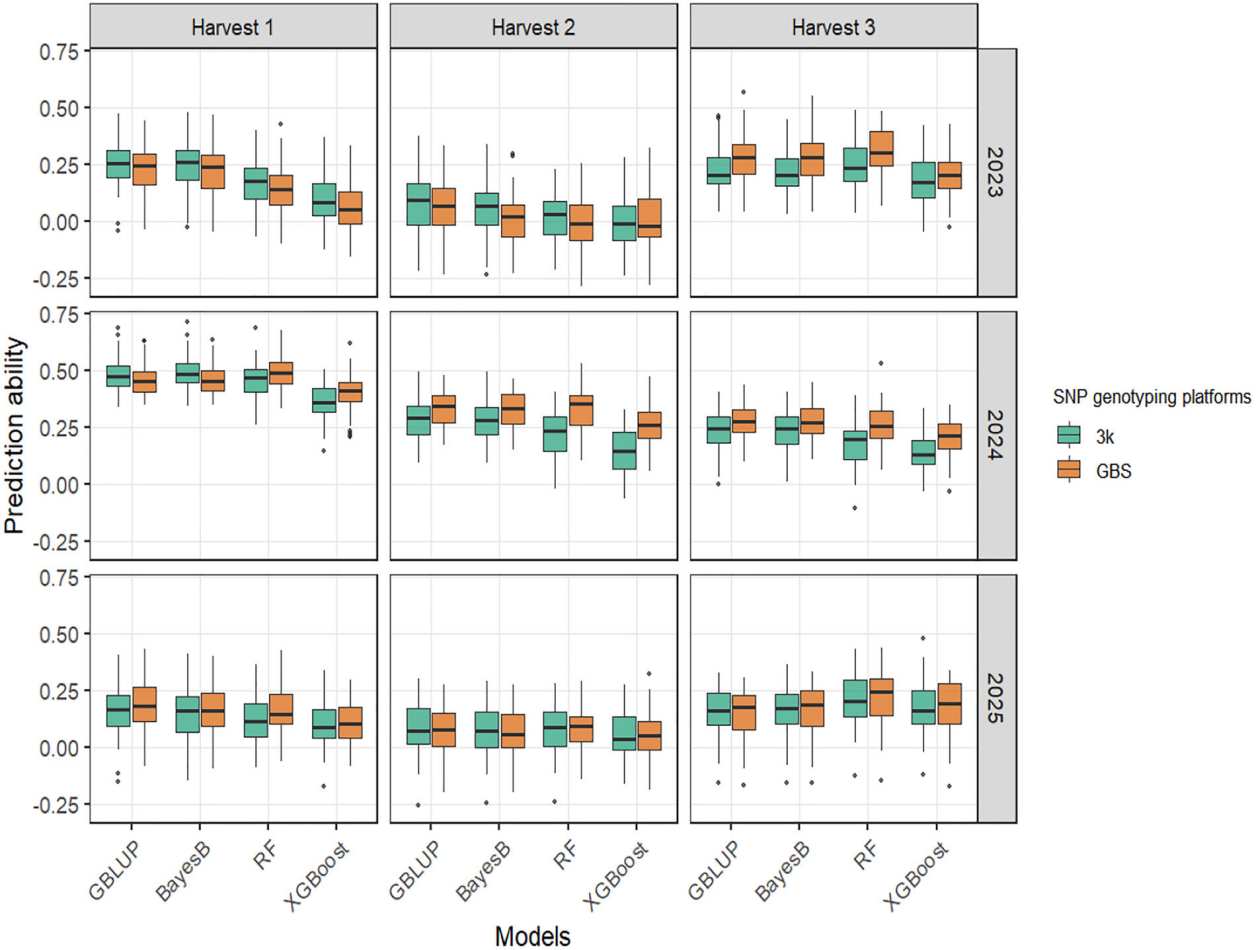
Predictive abilities of dry matter yield across harvests and years based on linear and machine learning models in cross validations. 3K, 3K single nucleotide platform (SNP) array‐based marker platform with1801 SNPs; GBLUP, genomic best linear unbiased prediction; GBS, genotyping by sequencing SNP marker platform with 12,415 SNPs; RF, random forest regression; XGBoost, extreme gradient boosting regression.

The genomic predictive ability of DMY was assessed utilizing fivefold cross‐validation for different statistical and machine learning models, including GBLUP, Bayes‐B, RF, and XGBoost with all the 12,415 GBS markers and 1801 3K array markers (Figure 5; Table S7). The genomic predictive abilities of different models obtained for the GBS and 3K marker platforms were comparable. The highest mean predictive abilities for DMY obtained using the GBLUP model were 0.480 and 0.461 for the 3K and GBS marker platforms, respectively, for harvest 1 (H1) in 2024. Other models had the highest predictive abilities for the same harvest period and year, followed by the second harvest period in 2024. In terms of comparison of model performance, the GBLUP, Bayes‐B, and RF models gave the highest and comparable predictive abilities for all harvests and years, as well as for the different marker platforms. The XGBoost method gave the lowest predictive ability in all harvests and years, with the highest predictive ability of 0.364 and 0.402 for harvest 1 (H1) in 2024 for the 3K and GBS platforms, respectively. For both genotyping platforms and all genomic prediction models, the second harvests in both years 2023 and 2025 had the lowest predictive abilities.

### 3.5 Multivariate genomic prediction

**FIGURE 6:**
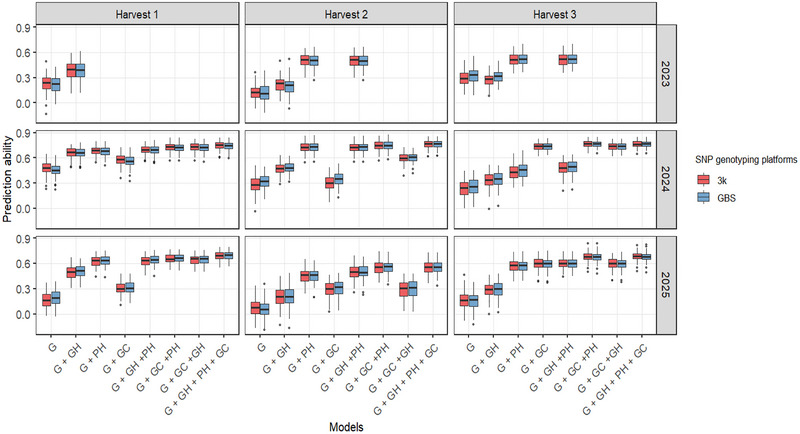
Mean predictive abilities of dry matter yield for univariate and multivariate genomic best linear unbiased prediction (GBLUP) prediction models with different combinations of secondary traits. 3K, 3K single nucleotide polymorphism (SNP) array‐based marker platform; G, genomic data; GBS, genotyping by sequencing SNP marker platform; GC, ground cover; GH, growth habit; H1, H2, and H3, biomass harvests 1–3; PH, plant height.

Multivariate genomic prediction was carried out using GBLUP and RF models, which were the best single‐trait statistical and machine learning models, respectively, in our study (Figures 6; Tables S8 and S9).

We tested the ability of multivariate genomic prediction models to predict DMY by utilizing secondary traits (PH, GH, and GC), which are easier to measure and have higher heritability, as covariate predictors in the multivariate genomic prediction models. There was a considerable improvement in the predictive ability of DMY with different combinations of the secondary traits used in the multivariate prediction models compared to the univariate models. The predictive ability increased as the number of traits used as covariates in the genomic prediction models increased. The model consisting of all three secondary traits and the primary trait (DMY) gave the highest predictive ability for both marker platforms in the different harvests and years. Considering the first harvest (H1) in the year 2024, which had the highest single trait mean predictive ability overall, the univariate model had predictive ability of 0.481, and the model which includes the three secondary traits (GH, GC, and PH) as predictors had predictive ability of 0.746 using 3K array markers (Figure 6; Table S8). Similarly, for the GBS SNPs, the univariate model showed predictive ability of 0.456, and the model involving GH, GC, and PH as predictors had predictive ability of 0.739 for DMY. These indicate comparable performance of both 3K and GBS marker platforms for the univariate and multivariate prediction of DMY. Similar trends were observed for both marker platforms in all harvests and years using the RF model for the univariate and multivariate genomic predictions (Table S9).

**FIGURE 7:**
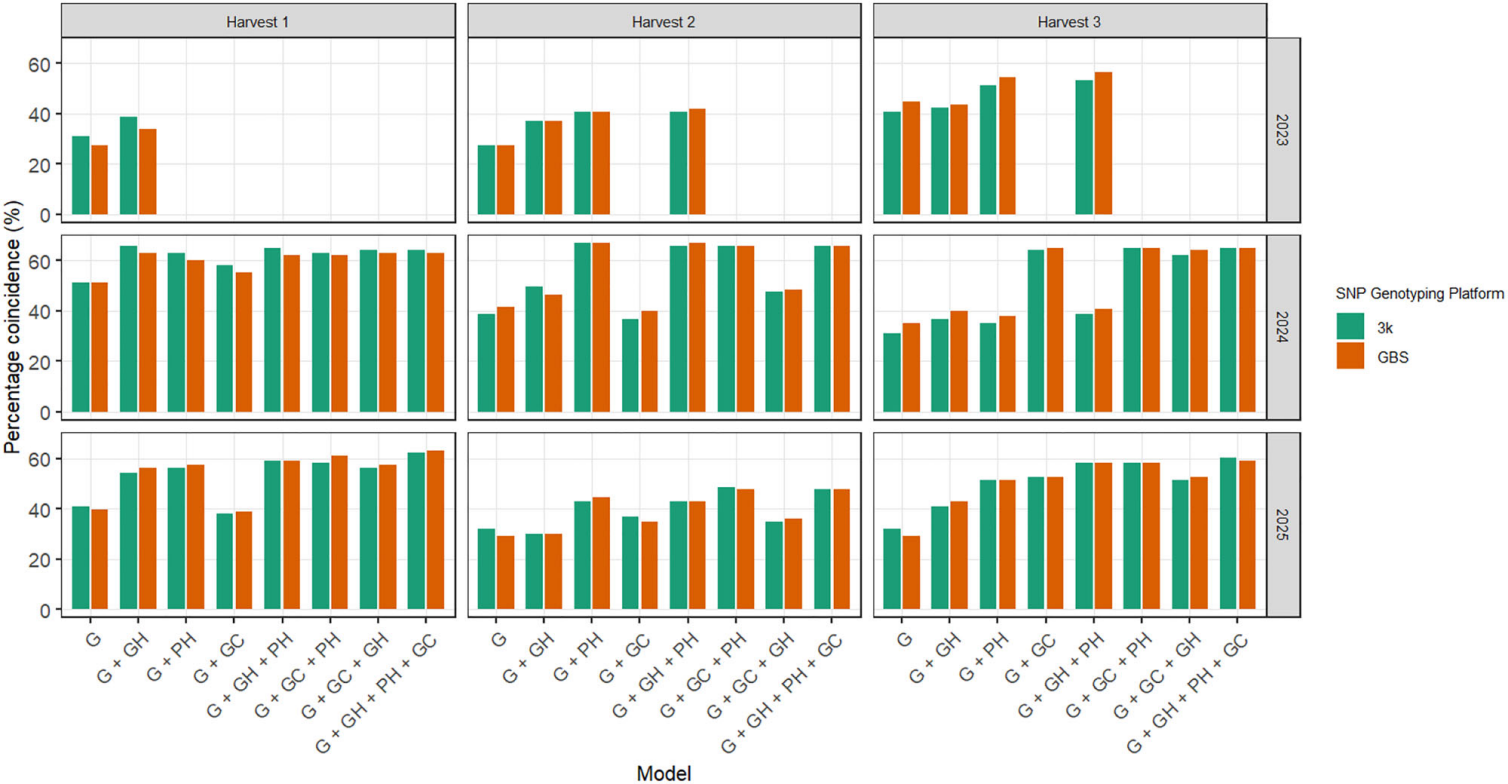
Comparison of the top 25% observed and predicted genotypes based on the genomic best linear unbiased prediction (GBLUP) univariate and multivariate models. 3K, 3K single nucleotide polymorphism (SNP) array‐based marker platform; G, genomic data; GBS, genotyping by sequencing SNP marker platform; GC, ground cover; GH, growth habit; H1, H2, and H3, biomass harvests 1–3; PH, plant height.

We also evaluated the percentage comparison of the top 25% observed and predicted genotypes based on GBLUP univariate and multivariate models. The percentage comparison increased as the predictive abilities of the models increased (Figure 7). The highest percentage overlaps (66.99%) were observed in the covariate models involving PH as a predictor, as well as in the model involving both GH and PH as covariate predictors in the second harvest (H2) in the year 2024.

The lowest percentage overlap between observed and predicted genotypes (27.11%) was observed for the univariate model in the second harvest (H2) in 2023.

### 3.6 Forward prediction of DMY across years and harvests

We utilized a forward prediction approach where genotype performance from harvests in earlier year(s) was used to predict DMY of the genotypes in later year harvests (Table 2). The predictive ability of DMY in the first harvests (H1) of subsequent years was the highest in all scenarios. The highest predictive ability was observed when DMY data from harvest 1 (H1) in the year 2023 were used to train the model to predict DMY of harvest 1 (H1) in the year 2024. This resulted in a comparable predictive ability of 0.306 and 0.301 using both 3K and GBS marker platforms, respectively. Similar results were obtained for both marker platforms in other scenarios. Combining harvest–year data for the years 2023 and 2024 to predict DMY in 2025 did not improve the predictive ability of the model. This resulted in a predictive ability of 0.100 for the 3K SNP array makers and 0.105 for the GBS SNP markers. The predictive ability of DMY in the second harvest (H2) based on the first harvest data was generally the highest in all scenarios. The highest predictive ability was observed when the DMY data obtained from the first harvest in 2024 were used to train the model and validated using the second harvest data in the same year, resulting in a predictive ability of 0.380 for the 3K array markers and 0.415 for the GBS SNP markers. The combination of the first and second harvest data to predict the performance of the lines in the third harvest in 2024 gave the highest predictive abilities of 0.187 and 0.167 for the 3K and GBS genotyping marker platforms, respectively, for this scenario. The forward prediction accuracies obtained in 2025 were relatively non‐informative, as predictive ability values were almost zero in all the scenarios.

**TABLE 2:** Forward predictive abilities of dry matter yield in different years within each harvest period using the two different marker platforms.

SNP assay	Year for training	Year for testing	Harvest	Predictive ability
3K	2023	2024	H1	0.306
			H2	0.256
			H3	0.084
	2024	2025	H1	0.261
			H2	0.025
			H3	0.151
	2023 and 2024	2025	H1	0.240
			H2	0.062
			H3	0.100
GBS	2023	2024	H1	0.301
			H2	0.260
			H3	0.103
	2024	2025	H1	0.270
			H2	0.004
			H3	0.150
	2023 and 2024	2025	H1	0.232
			H2	0.089
			H3	0.105

Abbreviations: 3K, 3K SNP array‐based marker platform; GBS, genotyping by sequencing SNP marker platform; H1, H2, and H3, biomass harvests 1–3; SNP, single nucleotide polymorphism.

The goal of the forward prediction was to be able to select for lines with superior DMY earlier in the season and years to reduce oat forage yield breeding cycle length. The forward predictive ability of DMY in different harvest periods within individual years was also examined using earlier harvest(s) data to predict subsequent harvest data (Table S10).

## 4 DISCUSSION

The cost, reproducibility, and speed of marker platforms for genotyping individuals to be selected for advancement have a substantial impact on the effectiveness of implementing GS in breeding programs. Konkolewska et al. (2023) found that designing SNP chips or comparable targeted genotyping platforms with minimal SNP density, containing uncorrelated SNPs, gave similar predictive abilities as using higher density SNP platforms. Reduction in marker density while retaining predictive ability is needed to develop breeder‐friendly genotyping assays for rapid and routine implementation in practical breeding programs.

### 4.1 Potential of low‐density marker platform for population structure patterns assessment

In this study, we used 409 oat lines from public breeding programs in the United States with multiple harvest–year phenotypic datasets to investigate the potential of array‐based and sequencing‐based SNP markers for univariate and trait‐assisted genomic prediction of biomass yield in oat breeding programs. Different marker platforms were also used to assess the population structure of the oat panel. These genotypes are mostly elite lines developed by small grains breeding programs in the Southern United States in the past years and have been used as parents for crosses by breeding programs in the region. For population structure, both 3K and GBS marker datasets identified similar genetic patterns in the panel and classified the genotypes into three clusters. The majority of the lines originated from the University of Florida and Louisiana State University breeding programs. The lack of substantial genetic diversity in the panel is because these breeding programs have exchanged germplasm for genetic improvement over the years. Similarly, Bazzer et al. (2025) reported no discrete clustering patterns from PCA of North American oat breeding lines.

### 4.2 Marker density and training population optimization using the 3K array‐ and GBS‐based SNP platforms

Several factors, such as the similarity between training and validation populations, training population size, marker number and genome coverage, trait heritability, extent of LD, trait complexity, marker platforms, as well as the interrelationship among these factors, influence predictive abilities of models in GS (Alemu et al., 2024; Covarrubias‐Pazaran et al., 2018). Marker density is one of the most studied factors influencing predictive abilities in GS. Higher number of markers typically results in greater predictive ability, attaining a peak depending on the trait architecture, the number of individuals in the training population, genome size, and LD (Berro et al., 2019; Covarrubias‐Pazaran et al., 2018; Sipowicz et al., 2025). Using both 3K and GBS marker platforms to assess the effect of marker density on the predictive ability of the different models, the observed predictive abilities were comparable. Using harvest 1 in year2024, which had the overall highest predictive ability, the predictive abilities obtained with a marker density of 20 were 0.276 and 0.335 for the 3K and GBS SNPs, respectively. We found an increase in predictive ability as the marker density increased from 20 to 1801, a point at which a plateau was reached by both marker platforms, resulting in a predictive ability percentage increase of 68.8% for the 3K dataset and 33.7% for the GBS dataset. More molecular markers are commonly useful for maintaining predictive ability over cycles of selection, ensuring a better picture of the LD pattern for capturing all quantitative trait loci (QTL) effects when the training population used is genetically diverse (Berro et al., 2019).

Likewise, increasing training population sizes resulted in increased predictive abilities of DMY. We found an increase in predictive ability with larger training population sizes, with peaks between 250 and 350 lines irrespective of the scenarios (marker platforms, year, and harvest combinations). Considering the DMY data obtained from the first harvest in 2024, the predictive abilities of DMY increased by 28.4% for the 3K SNPs and 43.6% for the GBS SNPs at a population size of 300. The predictive ability obtained with a training population size of 50 was 0.355 and 0.193 for the 3K and GBS SNPs, respectively. Several studies have proven that predictive ability is higher when larger training set sizes are used (Covarrubias‐Pazaran et al., 2018; Lozada et al., 2019; Zhu et al., 2021; Sørensen et al., 2023; Fernández‐González et al., 2023; Sipowicz et al., 2025).

### 4.3 Comparing the 3K array‐ and GBS‐based SNPs for DMY prediction

GS holds great potential for enabling the identification of superior lines with desirable biomass yield traits in oat forage breeding, unlike phenotypic selection, which is tedious, time‐consuming, and difficult to harness over large areas and in multiple locations (Maulana et al., 2019). The array‐ and GBS‐based SNPs were compared for the mean predictive abilities of DMY using four prediction models in cross‐validation. The GBLUP, Bayes‐B, and RF models gave similar predictive abilities for DMY, unlike the XGBoost model, which gave the lowest predictions in all cases. All the models performed similarly for both marker platforms. Yu et al. (2023) reported higher predictive abilities using GBS and target sequence markers compared to array‐based markers in 305 corn hybrids derived from 149 parental lines. They also found that XGBoost performed poorly in genomic prediction in all cases. The differences observed between XGBoost and other models may be due to the disparities in the models’ assumptions in the distribution of SNP effects (Elbasyoni et al., 2018). This may also be due to the presence of QTL with large effects segregating in the panel, which was better accounted for using Bayes‐B, and RF models (Rio et al., 2021), while the GBLUP model better accounted for several minor effects QTLs segregating in the panel, contributing additively to biomass yield (Barretto et al., 2024). Maulana et al. (2019) evaluated the prediction accuracies of ridge regression, best linear unbiased prediction, Gaussian kernel, and Bayesian least absolute shrinkage and selection operator models for forage quality traits of winter wheat using 289 genotypes and 90k SNPs. The authors reported similar prediction accuracy for the three models with values ranging from 0.34 to 0.61. Rio et al. (2021) assessed genomic prediction in a Mediterranean oat population consisting of 709 cultivated oat inbred lines with 17,288 biallelic SNP markers. They found similar predictive abilities for heading, PH, grain yield, and biomass using GBLUP, reproducing kernel Hilbert space, and multigroup GBLUP, except for RF.

### 4.4 Integrating secondary traits as covariate predictors for DMY prediction

The DMY is a highly quantitative trait controlled by several genes of small effects with lower heritability compared to secondary traits such as PH, GH, and GC, which are easier to measure. The moderate to high heritability estimates obtained for the evaluated secondary traits in our study suggest the possibility of exploring these easy‐to‐measure and highly heritable traits in multivariate GS approaches to improve DMY. Incorporating multiple traits in genomic prediction is very helpful when phenotypic data contain associated traits because information is borrowed between traits and among relatives (Gaire et al., 2022). The prediction accuracy of multivariate models is influenced by trait complexity, the relationship between training and test populations, secondary trait heritability, the statistical model used, and correlation between the primary and secondary traits (Dhakal et al., 2024). Multivariate models that included combinations of highly or moderately correlated secondary traits have been reported to give significantly or marginally higher predictive abilities than the univariate models in cross‐validation (Lozada & Carter, 2019; Sandhu et al., 2021; Zhang‐Biehn et al., 2021). In our present study, both the 3K array‐based and GBS‐derived SNPs gave similar predictive abilities for the multivariate genomic predictions using GBLUP and RF models. A higher increment in predictive abilities of the multivariate models was observed when PH was included in the model in the different harvests and years. This was particularly due to the high association between PH and DMY, as well as moderate to high heritability estimates of PH. Similarly, GH also had high heritability, and it showed high predictive ability for DMY in the model involving only GH as the secondary trait predictor in the first harvest (H1) of each year. This is because the GH data of the lines were only collected before the first biomass harvest in each year, after which the exact growth architecture of the genotype may not be accurately estimated due to the alteration in growth and development of the plants resulting from the subsequent harvests. The model involving genomic data plus all other easy‐to‐measure secondary traits as predictors resulted in the highest predictive abilities in all scenarios. This is due to the cumulative correlation of the secondary traits with DMY. This indicates the potential of multivariate genomic prediction to outperform the routine indirect selection of biomass yield using correlated secondary traits (Lozada & Carter, 2019). In general, the multivariate models performed better than the univariate GBLUP and RF models for DMY prediction in this study, resulting in more than twofold increases in predictive ability. Nonetheless, the extent of improvement of the multivariate models differs based on the different combinations of traits, harvests, and years of experiment. Fernandes et al. (2018) obtained a 50% increase in prediction accuracy when PH was used in both training and validation populations, along with the marker dataset to predict biomass yield in sorghum. Zhang‐Biehn et al. (2021) reported an improvement in prediction accuracy of end use quality traits using multivariate genomic prediction in 462 advanced hard winter wheat breeding lines using grain protein or flour protein as secondary trait predictors compared to the univariate models. For phenotyping of DMY in forage breeding programs on a large scale and in multiple environments, Arojju et al. (2020) recommended complete phenotyping of large training sets for secondary traits (which are comparatively less expensive and can be phenotyped on a large scale) and partly phenotyping primary trait (challenging and costly to assess), and then utilize multi‐trait prediction model to predict the primary trait of interest for selection. The higher overlap percentage found between the 25% top‐performing observed and predicted genotypes for multivariate GBLUP models (30.10%–66.99%) compared to the univariate model (27.18%–51.46%) further confirms the great potential of incorporating secondary trait predictors in genomic prediction models to improve genetic gains of forage yield in breeding programs. Our current multivariate genomic prediction approach will not eliminate the need for planting our test populations in the field, but it would avoid the need for manual or mechanical biomass harvesting of newly developed/previously untested genotypes to be predicted, which is the most challenging. Instead, secondary traits data plus genomic data of the new genotypes will be obtained and incorporated into our multivariate models to predict their biomass yield performance.

### 4.5 Forward prediction of DMY in future harvest and years

Forecasting the performance of unphenotyped genotypes for complex traits is usually challenging in breeding programs (Azizinia et al., 2023). Our results showed variation in the forward prediction of DMY for later harvest periods within the same year and the same harvest periods in different years, whereas the different marker platforms gave similar performance. For the same year harvest prediction, the highest predictive abilities were observed for DMY from the second biomass harvest (H2) predicted based on the first biomass harvest (H1) within the same year. When earlier year(s) harvest data were used to predict later year harvest data, DMY predictive abilities of the first biomass harvests (H1) were the highest. The observed low forward predictive ability of DMY for the second and third biomass harvests in the same year, as well as in future years, may be due to changes in the growth architecture of the plants after the first biomass cut, which alters PH, leaf‐to‐stem ratio, and forage yield. The low forward predictive abilities for future harvests and years obtained in our study agree with the findings of Jarquin et al. (2017), who reported the difficulty in predicting future years for grain yield in hard red winter wheat, which resulted in low prediction accuracy (*r* = 0.171) despite including environments with large population size. For future studies, the inclusion of high‐throughput phenotyping and environmental covariates information could further enhance the predictive ability of DMY in later harvests and years.

In terms of predictive abilities, both the 3K SNP array and GBS marker datasets gave comparable results. Since the reproducibility, speed, and ease of production of molecular markers, as well as computational cost, cannot be ignored in practical breeding programs, the 3K SNP array marker dataset with much lower marker density could be a marker of choice for population structure and genomic prediction analyses, depending on the goal of the research. Our findings corroborate those of Bazzer et al. (2025), who reported that the lower number of the USDA‐ARS 3K array markers did not reduce prediction accuracy compared to using GBS SNP markers. Our findings in the present study would assist breeders in making informed decisions when choosing genotyping platforms for genetic diversity and genomic prediction analyses. C. Liu et al. (2020) recommended the use of very high‐density and medium‐density marker platforms for pure gene discovery to ensure sufficient power to identify marker trait association from genome‐wide association studies and for GS. Overall, our results suggest that the selection of candidate individuals in early generations using multivariate GS with the robust 3K array markers could be leveraged by breeders to shorten the length of the breeding cycle and thus increase genetic gain in oat forage breeding programs.

## 5 CONCLUSIONS

We compared two different marker platforms for assessing population structure patterns, the effects of marker density and varying number of individuals, as well as univariate and multivariate models on genomic predictive abilities. Our study showed similar performance of 3K array‐ and GBS‐based SNPs in terms of genetic diversity, marker density, and training population optimization, as well as single and multiple trait genomic prediction of forage yield. There was an increase in predictive ability as marker number and training population sizes increased, with peaks at 250–350 genotypes irrespective of the scenarios (marker platforms, year, and harvest combination) and plateaued for marker density at 1801 markers. The multivariate prediction models showed higher predictive ability for DMY than the univariate models. The observed improvement in predictive ability was mainly due to the correlation between DMY and other secondary traits, as well as the higher heritability of the secondary traits. The reduced number of markers in the 3K array‐based platform compared to GBS‐based SNPs did not result in a reduction in prediction accuracy. The cost‐effectiveness and less computational resources required for the 3K array platform would enhance its utilization for genomic prediction, particularly in the early stages of a breeding program, where breeders have to screen and select from a large number of genotypes. Incorporating high‐throughput phenotyping data is a cost‐effective strategy to further improve the prediction accuracy of oat forage yield on a large scale and in multiple environments to further speed up the rate of genetic gain of forage yield in breeding programs.

## AUTHOR CONTRIBUTIONS

**Samuel A. Adewale**: Conceptualization; data curation; formal analysis; investigation; methodology; writing—original draft. **Md Ali Babar**: Conceptualization; funding acquisition; investigation; methodology; resources; writing—review and editing. **Diego Jarquin**: Formal analysis; writing—review and editing. **Naeem Khan**: Investigation; writing—review and editing. **Janam Prabhat Acharya**: Investigation; writing—review and editing. **Sudip Kunwar**: Investigation; writing—review and editing. **Jordan McBreen**: Investigation; writing—review and editing. **Esteban Rios**: Resources; writing—review and editing. **Stephen Harrison**: Resources; writing—review and editing. **Noah DeWitt**: Resources; writing—review and editing. **Amir Ibrahim**: Resources; writing—review and editing. **Paul Murphy**: Resources; writing—review and editing. **Rick Boyles**: Resources; writing—review and editing. **Jason D. Fiedler**: Data curation; resources; writing—review and editing. **Raja Sekhar Nandety**: Data curation; writing—review and editing.

## CONFLICT OF INTEREST STATEMENT

The authors declare no conflicts of interest.

## Supporting information



Supplementary Figure S1. Optimal number of clusters for population structure in the SOAP panel using 1,801 SNPs from the 3K marker platform (A) and 12,415 GBS‐based SNP markers (B).
Supplementary Figure S2. K‐means clustering showing population structure in the SOAP panel based on 1,801 SNPs from the 3K marker platform (A) and 12,415 GBS‐based SNP markers (B).
Supplementary Figure S3. Histogram showing distribution of phenotypic traits measured in 2023. GH—growth habit, PH_H2 and PH_H3 – plant height for harvests 2 and 3 respectively, DMY_H1, DMY_H2 and DM_H3 – dry matter yield for harvests 1, 2 and 3 respectively. Ground cover and plant height data at harvest 1 were not collected in 2023.
Supplementary Figure S4. Histogram showing distribution of phenotypic traits measured in 2024. GH—growth habit, GC_H1, GC_H2 and GC _H3 – ground cover for harvests 1, 2 and 3 respectively, PH_H1, PH_H2 and PH_H3 – plant height for harvests 1, 2 and 3 respectively, DMY_H1, DMY_H2 and DM_H3 – dry matter yield for harvests 1, 2 and 3 respectively.
Supplementary Figure S5. Histogram showing distribution of phenotypic traits measured in 2025. GH—growth habit, GC_H1, GC_H2 and GC _H3 – ground cover for harvests 1, 2 and 3 respectively, PH_H1, PH_H2 and PH_H3 – plant height for harvests 1, 2 and 3 respectively, DMY_H1, DMY_H2 and DM_H3 – dry matter yield for harvests 1, 2 and 3 respectively.
Supplementary Figure S6. Pearson correlation between primary and secondary traits for harvests 1 – 3 in 2023 growing season. H1, H2, and H3 – biomass harvests 1 – 3, GH – growth habit, PH – plant height, DMY – dry matter yield. Plant height data before the first harvest and ground cover data were not collected in the year 2023.
Supplementary Figure S7. Pearson correlation between primary and secondary traits for harvests 1 – 3 in the 2024 growing season. H1, H2, and H3 – biomass harvests 1 – 3, GH – growth habit, PH – plant height, GC – ground cover, DMY – dry matter yield.
Supplementary Figure S8. Pearson correlation between primary and secondary traits for harvests 1 – 3 in the 2025 growing season. H1, H2, and H3 – biomass harvests 1 – 3, GH – growth habit, PH – plant height, GC – ground cover, DMY – dry matter yield.

Supplementary Table S1. List of 420 Southern Oat Association Panel lines used in the study.
Supplementary Table S2. Mantel test comparing the relationship matrices derived from 3K and GBS marker platforms.
Supplementary Table S3. Descriptive statistics of biomass‐related traits from three harvests each in three years.
Supplementary Table S4. Heritability of biomass‐related traits for each harvest and across environments.
Supplementary Table S5. Predictive abilities of dry matter yield using varying population sizes in different harvest periods and years.
Supplementary Table S6: Effects of varying marker numbers on prediction accuracies of dry matter yield comparing GBS and 3k marker platforms.
Supplementary table S7. Predictive abilities of dry matter yield across harvests and years based on linear and machine learning models in cross validations.
Supplementary Table S8. Mean predictive abilities of dry matter yield for univariate and multivariate GBLUP prediction models with different combinations of secondary traits.
Supplementary Table S9. Mean predictive abilities of dry matter yield for univariate and multivariate random forest prediction models with different combinations of secondary traits.
Supplementary Table S10. Forward predictive abilities of dry matter yield in different harvest periods within each year.

## Data Availability

All data generated or analyzed during this study are available at https://github.com/SAdewale75/Covariate_GP.git
