# Supplementary material for: Effectiveness of low‐density high‐throughput marker platform and easy‐to‐measure traits for genomic prediction of biomass yield in oat (Avena sativa L.)
Source: Plant Genome. 2026 Jan 16;19(1):e70179. doi: 10.1002/tpg2.70179 (PMC12823034; doi:10.1002/tpg2.70179)
Supplement: Supplementary file 1 [file TPG2-19-e70179-s001.docx]

**Effectiveness of low-density high-throughput marker platform and easy-to-measure traits for genomic prediction of biomass yield in oat (*Avena sativa* L.).**

**Samuel A. Adewale^1,2^, Md Ali Babar ^2*^, Diego Jarquin^2^, Naeem Khan^2^, Janam Prabhat Acharya^2^, Sudip Kunwar^1,2^, Jordan McBreen^2^, Esteban Rios^2^, Stephen Harrison^3^, Noah DeWitt^3^, Amir Ibrahim^4^, Paul Murphy^5^, Rick Boyles^6^, Jason D. Fiedler^7^, Raja Sekhar Nandety^7^**

^1^Plant Breeding Graduate Program, University of Florida, Gainesville, Florida, USA

^2^Department of Agronomy, University of Florida, Gainesville, Florida, USA

^3^School of Plant, Environmental and Soil Sciences, Louisiana State University, Baton Rouge, LA 70803, USA.

^4^AgriLife Research, Texas A&M University, College Station, TX 77843, USA.

^5^Department of Crop and Soil Sciences, North Carolina State University, Raleigh, NC 27695, USA.

^6^Pee Dee Research and Education Center, Clemson University, Florence, SC 29506, USA.

^7^ Cereal Crops Improvement Research Unit, Edward T. Schafer Agricultural Research Center, USDA-ARS, Fargo, ND 58102, USA.

**Correspondence**

*Md Ali Babar, Department of Agronomy, University of Florida, 3105 McCarty Hall B,

Gainesville, FL 32608, USA. Email: mababar@ufl.edu

Number of pages - 56

Number of figures - 7

Number of tables - 2

Supplementary Figure S1. Optimal number of clusters for population structure in the SOAP panel using 1,801 SNPs from the 3K marker platform (A) and 12,415 GBS-based SNP markers (B).

Supplementary Figure S2. K-means clustering showing population structure in the SOAP panel based on 1,801 SNPs from the 3K marker platform (A) and 12,415 GBS-based SNP markers (B).

Supplementary Figure S3. Histogram showing distribution of phenotypic traits measured in 2023. GH - growth habit, PH_H2 and PH_H3 – plant height for harvests 2 and 3 respectively, DMY_H1, DMY_H2 and DM_H3 – dry matter yield for harvests 1, 2 and 3 respectively. Ground cover and plant height data at harvest 1 were not collected in 2023.

Supplementary Figure S4. Histogram showing distribution of phenotypic traits measured in 2024. GH - growth habit, GC_H1, GC_H2 and GC _H3 – ground cover for harvests 1, 2 and 3 respectively, PH_H1, PH_H2 and PH_H3 – plant height for harvests 1, 2 and 3 respectively, DMY_H1, DMY_H2 and DM_H3 – dry matter yield for harvests 1, 2 and 3 respectively.

Supplementary Figure S5. Histogram showing distribution of phenotypic traits measured in 2025. GH - growth habit, GC_H1, GC_H2 and GC _H3 – ground cover for harvests 1, 2 and 3 respectively, PH_H1, PH_H2 and PH_H3 – plant height for harvests 1, 2 and 3 respectively, DMY_H1, DMY_H2 and DM_H3 – dry matter yield for harvests 1, 2 and 3 respectively.

Supplementary Figure S6. Pearson correlation between primary and secondary traits for harvests 1 – 3 in 2023 growing season. H1, H2, and H3 – biomass harvests 1 – 3, GH – growth habit, PH – plant height, DMY – dry matter yield. Plant height data before the first harvest and ground cover data were not collected in the year 2023.

Supplementary Figure S7. Pearson correlation between primary and secondary traits for harvests 1 – 3 in the 2024 growing season. H1, H2, and H3 – biomass harvests 1 – 3, GH – growth habit, PH – plant height, GC – ground cover, DMY – dry matter yield.

Supplementary Figure S8. Pearson correlation between primary and secondary traits for harvests 1 – 3 in the 2025 growing season. H1, H2, and H3 – biomass harvests 1 – 3, GH – growth habit, PH – plant height, GC – ground cover, DMY – dry matter yield.
