# Supplementary material for: Effectiveness of low‐density high‐throughput marker platform and easy‐to‐measure traits for genomic prediction of biomass yield in oat (Avena sativa L.)
Source: Plant Genome. 2026 Jan 16;19(1):e70179. doi: 10.1002/tpg2.70179 (PMC12823034; doi:10.1002/tpg2.70179)
Supplement: Supplementary file 2 [file TPG2-19-e70179-s002.docx]

**Effectiveness of low-density high-throughput marker platform and easy-to-measure traits for genomic prediction of biomass yield in oat (*Avena sativa* L.).**

**Samuel A. Adewale^1,2^, Md Ali Babar ^2*^, Diego Jarquin^2^, Naeem Khan^2^, Janam Prabhat Acharya^2^, Sudip Kunwar^1,2^, Jordan McBreen^2^, Esteban Rios^2^, Stephen Harrison^3^, Noah DeWitt^3^, Amir Ibrahim^4^, Paul Murphy^5^, Rick Boyles^6^, Jason D. Fiedler^7^, Raja Sekhar Nandety^7^**

^1^Plant Breeding Graduate Program, University of Florida, Gainesville, Florida, USA

^2^Department of Agronomy, University of Florida, Gainesville, Florida, USA

^3^School of Plant, Environmental and Soil Sciences, Louisiana State University, Baton Rouge, LA 70803, USA.

^4^AgriLife Research, Texas A&M University, College Station, TX 77843, USA.

^5^Department of Crop and Soil Sciences, North Carolina State University, Raleigh, NC 27695, USA.

^6^Pee Dee Research and Education Center, Clemson University, Florence, SC 29506, USA.

^7^ Cereal Crops Improvement Research Unit, Edward T. Schafer Agricultural Research Center, USDA-ARS, Fargo, ND 58102, USA.

**Correspondence**

*Md Ali Babar, Department of Agronomy, University of Florida, 3105 McCarty Hall B,

Gainesville, FL 32608, USA. Email: mababar@ufl.edu

Number of pages - 56

Number of figures - 7

Number of tables - 2

Supplementary Table S1. List of 420 Southern Oat Association Panel lines used in the study.

| Entry no | Entry name |
| --- | --- |
| 1 | BIG MAC |
| 2 | BOB |
| 3 | BROOKS |
| 4 | Caballo |
| 5 | Cantara |
| 6 | COKER 227 |
| 7 | COKER 234 |
| 8 | Coker 716 |
| 9 | Corondo |
| 10 | FL0047-J9 |
| 11 | FL0115-J2 |
| 12 | FL03001BSB-S7 |
| 13 | FL03146BSB-S1-B-S1 |
| 14 | FL03254-L1 |
| 15 | FL04155-S06-31-B-S1 |
| 16 | FL0720-R6 |
| 17 | FL0772-R3 |
| 18 | FL0914-U2 |
| 19 | FL0923-U2 |
| 20 | FL0923-U4 |
| 21 | FL0941-U1 |
| 22 | FL0943-U2 |
| 23 | FL0943-U4 |
| 24 | FL1013-1 |
| 25 | FL1013-2 |
| 26 | FL1035-2 |
| 27 | FL1045-1 |
| 28 | FL1049-3 |
| 29 | FL11044-1 |
| 30 | FL99084-J2 |
| 31 | FL99153FBS-45-1-B-S-B-S1-B-S1 |
| 32 | FLORIDA 02011 |
| 33 | Florida 500 |
| 34 | FLORIDA 501 |
| 35 | FLORIDA 502 |
| 36 | Fulgrain |
| 37 | Gerard 224 |
| 38 | Gerard 229 |
| 39 | Harrison |
| 40 | Horizon 201 |
| 41 | HORIZON 270 |
| 42 | HORIZON 306 |
| 43 | LA02012-S-B-139-S2-B-S2-B-S2 |
| 44 | LA02035-I-J1 |
| 45 | LA02065SBSBSBSB-88 |
| 46 | LA03018SBSBSB-65-S1 |
| 47 | LA03040SBSBSB-83 |
| 48 | LA03046SBS7-B-S1 |
| 49 | LA03060SBSBSB-S1 |
| 50 | LA03066SBS-L1 |
| 51 | LA04004SBSB-121 |
| 52 | LA04004SBSB-61-B-S1 |
| 53 | LA04004SBSB-7-B-S1 |
| 54 | LA04014SBSB-39 |
| 55 | LA04018SBSB-181 |
| 56 | LA04018SBSB-86 |
| 57 | LA05011GSBS-30 |
| 58 | LA05011GSBSBSB-11 |
| 59 | LA06009SBSBSB-24-S |
| 60 | LA06046-N2-Ab3 |
| 61 | LA06058SBS-32-S1 |
| 62 | LA06059SBS-84-S1 |
| 63 | LA06059SBSBSB-16-S |
| 64 | LA06059SBSBSB-97-S |
| 65 | LA06059SBSBSBSB-4-S1 |
| 66 | LA06063SBSB-S1 |
| 67 | LA06063SBSBSB-13 |
| 68 | LA07005SBSBSB-12 |
| 69 | LA07007SBSB-68 |
| 70 | LA07007SBSBSB-18 |
| 71 | LA07007SBSBSB-24 |
| 72 | LA07017SBSB-30 |
| 73 | LA07023SBS-FS5-Ab1 |
| 74 | LA07024SBSBSBS-7-S1 |
| 75 | LA07026SBS-FS1-Ab1 |
| 76 | LA07033SBSB-2 |
| 77 | LA07033SBSB-33 |
| 78 | LA07046SBSBSBS-2-S1 |
| 79 | LA07048SBSB-19 |
| 80 | LA07048SBSB-28 |
| 81 | LA07048SBSB-39 |
| 82 | LA07048SBSB-5 |
| 83 | LA07049SBSBSBS-52-S1 |
| 84 | LA07052SBSBSBS-1-S1 |
| 85 | LA07059SBSBSB-44 |
| 86 | LA07065SBSBSB-32 |
| 87 | LA07065SBS-FS2-Ab1 |
| 88 | LA07070SBSBSBS-65-S1 |
| 89 | LA08016SBSBS-44-S1 |
| 90 | LA08021SBSBS-15-S1 |
| 91 | LA08021SBSBS-7-S1 |
| 92 | LA08024SBSBS-29-S1 |
| 93 | LA08024SBSBS-48-S1 |
| 94 | LA08039SBSBS-5-S1 |
| 95 | LA08051SBSB-32 |
| 96 | LA08056SBSBS-56-S1 |
| 97 | LA08081BS-T2 |
| 98 | LA08083SBSBS-5 |
| 99 | LA08084SBSBS-15 |
| 100 | LA08085BS-T1 |
| 101 | LA08085BS-T2 |
| 102 | LA08085BS-T4 |
| 103 | LA08085SBSBS-48 |
| 104 | LA08085SBSBSB-48 |
| 105 | LA08085SS-T3 |
| 106 | LA08086BS-T2 |
| 107 | LA08086SBSBS-34 |
| 108 | LA08089SBSBS-52-S1 |
| 109 | LA09013SBS-U4 |
| 110 | LA09013SBS-U6 |
| 111 | LA09015SBS-U1 |
| 112 | LA09016SBS-U6 |
| 113 | LA09026SBS-U3 |
| 114 | LA09028SBS-U2 |
| 115 | LA09040SBS-U1 |
| 116 | LA09045SBS-U3 |
| 117 | LA09065SBS-U1 |
| 118 | LA09066SBS-U3 |
| 119 | LA09067SBS-U1 |
| 120 | LA09078SBS-U5 |
| 121 | LA09078SBS-U7 |
| 122 | LA09082SBS-U11 |
| 123 | LA09092SBS-U1 |
| 124 | LA09092SBS-U2 |
| 125 | LA09103SBS-U4 |
| 126 | LA09103SBS-U5 |
| 127 | LA10044-4 |
| 128 | LA9339 (PLOT SPIKE, RAM OAT) |
| 129 | LA97006GSB-59-2-4-SBS1 |
| 130 | LA99016 |
| 131 | LA99017 (BUCK) |
| 132 | NC09-4274N |
| 133 | NC10-5069y |
| 134 | NC11-1798y |
| 135 | NC12-3447 |
| 136 | NC12-3578 |
| 137 | NC12-3742 |
| 138 | NC12-3753 |
| 139 | NC12-3845 |
| 140 | NC12-3872 |
| 141 | NC12-3922 |
| 142 | NC12-3931 |
| 143 | NC12-3963 |
| 144 | NC13-6579 |
| 145 | NC13-6584 |
| 146 | NC13-6589 |
| 147 | NC14-1482 |
| 148 | NC14-1491 |
| 149 | NC14-1494 |
| 150 | NC14-1498 |
| 151 | NC14-1501 |
| 152 | NC14-1514 |
| 153 | NC14-1533 |
| 154 | NC14-1543 |
| 155 | NC14-1554 |
| 156 | NC14-1573 |
| 157 | NC14-1574 |
| 158 | NC14-1584 |
| 159 | NC14-1605 |
| 160 | NC14-1609 |
| 161 | NC14-1619 |
| 162 | NC14-1643 |
| 163 | NC14-1651 |
| 164 | NC14-1686 |
| 165 | NC14-1695 |
| 166 | NC14-1747 |
| 167 | NC14-1751 |
| 168 | NC14-1767 |
| 170 | NC14-1787 |
| 171 | NC14-1792 |
| 172 | NC14-1799 |
| 173 | NC14-1818 |
| 174 | NC14-1820 |
| 175 | NC14-1832 |
| 176 | NC14-1874 |
| 177 | NC14-1902 |
| 178 | NC14-1965 |
| 179 | NC14-1974 |
| 180 | NC14-1977 |
| 181 | NC14-1985 |
| 182 | NC14-1989 |
| 183 | Osage |
| 184 | Ozark |
| 185 | Rodgers |
| 186 | SECTARIAT LA495 |
| 187 | Simpson |
| 188 | SS76-50 |
| 189 | TAMO 405 |
| 190 | TAMO 406 |
| 191 | TAMO 606 |
| 192 | TIFT |
| 193 | TROPHY |
| 194 | TX02U 7047 |
| 195 | TX02U 7097 |
| 196 | TX02U 7473 |
| 197 | TX02U 7479 |
| 198 | TX02U 7605 |
| 199 | TX05CS 347-1 (??TAMO405) |
| 200 | TX05CS 347-2 |
| 201 | TX07CS1965 |
| 202 | TX07CS2140 |
| 203 | TX07CS2652 |
| 204 | TX07CS2765 |
| 205 | TX09CS058 |
| 206 | TX09CS1056 |
| 207 | TX09CS1112 |
| 208 | TX10CAS085 |
| 209 | TX14OCS5004 |
| 210 | TX14OCS5010 |
| 211 | TX14OCS5011 |
| 212 | TX14OCS5012 |
| 213 | TX14OCS5014 |
| 214 | TX14OCS5017 |
| 215 | TX14OCS5018 |
| 216 | TX14OCS5031 |
| 217 | TX14OCS5036 |
| 218 | TX14OCS5037 |
| 219 | TX14OCS5039 |
| 220 | TX14OCS5041 |
| 221 | TX14OCS5050 |
| 222 | TX14OCS5054 |
| 223 | TX14OCS5061 |
| 224 | TX14OCS5065 |
| 225 | TX14OCS5069 |
| 226 | TX14OCS5070 |
| 227 | TX14OCS5078 |
| 228 | TX14OCS5080 |
| 229 | TX14OCS5098 |
| 230 | TX14OCS5112 |
| 231 | TX14OCS5116 |
| 232 | TX14OCS5182 |
| 233 | TX14OCS5193 |
| 234 | Winter Fulghum |
| 235 | FLLA11019S-8 |
| 236 | TAMO412 |
| 237 | FL12034-10 |
| 238 | FL11017-7 |
| 239 | FLLA09044SBS-U1 |
| 240 | FL13057-4 |
| 241 | FL13088-3 |
| 242 | FL13088-4 |
| 243 | FL13097-1 |
| 244 | FL13084-8 |
| 245 | LA09015SBS-U4 |
| 246 | FLLA13043-57 |
| 247 | FL13108-62 |
| 248 | FL13109-38 |
| 249 | FL13110-68 |
| 250 | FL13112-14 |
| 251 | FL13113-69 |
| 252 | FL13123-41 |
| 253 | FL13123-50 |
| 254 | FL13123-54 |
| 255 | FL13123-60 |
| 256 | FL13123-73 |
| 257 | FL13125-17 |
| 258 | FL13126-57 |
| 259 | FL13126-69 |
| 260 | FL13126-79 |
| 262 | FLLA17028-12 |
| 264 | FLLA17055-63 |
| 265 | FLLA17067-12 |
| 267 | FLLA17071-4 |
| 268 | FLLA17074-47 |
| 269 | FLLA17077-4 |
| 271 | FLLA17088-60 |
| 272 | FLLA17100-4 |
| 273 | FLLA17109-56 |
| 275 | FLLA17119-74 |
| 276 | FLLA17149-1 |
| 277 | FLLA17152-47 |
| 280 | FLLA17030-18 |
| 282 | FLLA17035-50 |
| 283 | FLLA17047-55 |
| 284 | FLLA17059-4 |
| 285 | FLLA17071-51 |
| 286 | FLLA17084-28 |
| 287 | FLLA17111-1 |
| 288 | FLLA16028-42 |
| 289 | FLLA16049-68 |
| 290 | FLLA16083-3 |
| 291 | FLLA16117-4 |
| 292 | FLLA16137-1 |
| 294 | FLLA16179-1 |
| 296 | FLLA16056-4 |
| 297 | FLLA16102-41 |
| 298 | FLLA16102-75 |
| 299 | FLLA16102-76 |
| 300 | FLLA16102-78 |
| 301 | TX20CAS1001 |
| 302 | TX20CAS1002 |
| 309 | TX20CAS1037 |
| 310 | TX20CAS1039 |
| 311 | TX20CAS1070 |
| 315 | TX20CAS1082 |
| 316 | LA15029SB-18-1-1 |
| 317 | LA17031SBS-58-1 |
| 318 | LA17076SBS-32-1 |
| 319 | LA17089SBS-25-2 |
| 320 | LA17089SBS-33-2 |
| 321 | LA14009SBSB-21-1-1 |
| 322 | LA16072SBSS-30-1 |
| 323 | LA17008SBSS-24-1 |
| 324 | LA17008SBSS-59-1 |
| 325 | LA17069SBSS-2-1 |
| 326 | LA17075SBSS-24-1 |
| 327 | LA17075SBSS-29-1 |
| 328 | LA17088SBSS-15-1 |
| 329 | LA17089SBS-45-1-1 |
| 330 | LA17089SBSS-73-1 |
| 331 | LA17110SBSS-16-1 |
| 332 | LA17129SBSS-60-1 |
| 333 | LA17129SBSS-8-1 |
| 334 | LA17153SBS-20-2-1 |
| 335 | LA17153SBSS-46-1 |
| 336 | LA17153SBSS-55-1 |
| 337 | FLLA17069-44 |
| 338 | FLLA17069-53 |
| 339 | FLLA17069-65 |
| 340 | FLLA17069-54 |
| 341 | FLLA17069-52 |
| 342 | FLLA17071-19 |
| 343 | FLLA17071-7 |
| 344 | FLLA17081-23 |
| 345 | FLLA17088-61 |
| 346 | FLLA17088-77 |
| 347 | FLLA17119-66 |
| 348 | FLLA17149-30 |
| 349 | FLLA17152-42 |
| 350 | FLLA17030-23 |
| 351 | FLLA16083-25 |
| 352 | TX19CAS0002 |
| 353 | TX19CAS0004 |
| 354 | TX19CAS0017 |
| 355 | TX19CAS0020 |
| 356 | TX19CAS0032 |
| 357 | TX19CAS0041 |
| 358 | TX19CAS0047 |
| 359 | TX19CAS0050 |
| 360 | TX19CAS0055 |
| 361 | TX19CAS0057 |
| 362 | TX19CAS0058 |
| 363 | TX19CAS0066 |
| 364 | TX19CAS0067 |
| 365 | TX19CAS0073 |
| 366 | TX19CAS0091 |
| 367 | SCLA17080SBS-4-2 |
| 368 | SCLA17152SBS-1-1 |
| 369 | SCLA16032SSBS-7-1 |
| 370 | SCLA17048SBS-4-2 |
| 371 | SCLA17119SBS-17-2 |
| 372 | SCLA17076SBS-32-1 |
| 373 | SCLA17119SBS-4-2 |
| 374 | SCLA17074SBS-1-1 |
| 375 | SCLA17048SBS-16-2 |
| 376 | SCLA17151SBS-40-1 |
| 377 | FLLA17025-4 |
| 378 | FLLA17025-20 |
| 379 | FLLA17029-66 |
| 380 | FLLA17029-75 |
| 381 | FLLA17032-41 |
| 382 | FLLA17051-2 |
| 383 | FLLA17051-20 |
| 384 | FLLA17051-21 |
| 385 | FLLA17067-35 |
| 386 | FLLA17069-41 |
| 387 | FLLA17069-49 |
| 388 | FLLA17069-56 |
| 389 | FLLA17069-73 |
| 390 | FLLA17069-76 |
| 391 | FLLA17077-16 |
| 392 | FLLA17077-28 |
| 393 | FLLA17078-54 |
| 394 | FLLA17088-66 |
| 395 | FLLA17100-5 |
| 396 | FLLA17109-68 |
| 397 | FLLA17149-3 |
| 398 | FLLA17152-71 |
| 399 | FLLA17158-11 |
| 400 | FLLA17158-18 |
| 401 | FLLA17158-30 |
| 402 | FLLA17017-69 |
| 403 | FLLA17017-70 |
| 404 | FLLA17018-17 |
| 405 | FLLA17030-25 |
| 406 | FLLA17031-57 |
| 407 | FLLA17031-71 |
| 408 | FLLA17032-5 |
| 409 | FLLA17035-69 |
| 410 | FLLA16166-79 |
| 411 | FLLA16179-3 |
| 412 | FLLA16179-9 |
| 413 | FLLA16180-50 |
| 414 | FLLA16056-12 |
| 415 | FLLA16056-25 |
| 416 | TX20CAS1006 |
| 417 | TX20CAS1011 |
| 418 | TX20CAS1013 |
| 419 | TX20CAS1014 |
| 420 | TX20CAS1021 |
| 421 | TX20CAS1024 |
| 422 | TX20CAS1025 |
| 423 | TX20CAS1034 |
| 424 | TX20CAS1035 |
| 425 | TX20CAS1041 |
| 426 | TX20CAS1044 |
| 427 | TX20CAS1046 |
| 428 | TX20CAS1047 |
| 429 | TX20CAS1048 |
| 430 | TX20CAS1056 |
| 431 | TX20CAS1061 |
| 432 | TX20CAS1062 |
| 433 | TX20CAS1063 |
| 434 | TX20CAS1071 |
| 435 | TX20CAS1074 |
| 436 | TX20CAS1075 |
| 437 | TX20CAS1076 |
| 438 | TX20CAS1077 |
| 439 | TX20CAS1079 |
| 440 | TX20CAS1080 |

Supplementary Table S2. Mantel test comparing the relationship matrices derived from 3K and GBS marker platforms.

| Marker platforms | Pearson correlation | P-value |
| --- | --- | --- |
| GBS vs 3K array | 0.7983 | 0.001 |

GBS – genotyping by sequencing-based marker platform, 3K array – 3K SNP array-based marker platform.

Supplementary Table S3. Descriptive statistics of biomass-related traits from three harvests each in three years.

| Trait | Year | Harvest | Min | Max | Mean | SD |
| --- | --- | --- | --- | --- | --- | --- |
| Growth habit | 2023 | H1 | 2.00 | 8.50 | 4.70 | 1.10 |
| Plant height, cm |  | H2 | 20.98 | 55.91 | 37.82 | 6.55 |
|  |  | H3 | 37.77 | 89.20 | 62.31 | 10.26 |
| DMY, kg/ha |  | H1 | 143.58 | 2893.01 | 1431.71 | 630.23 |
|  |  | H2 | 233.95 | 5779.53 | 3472.76 | 1125.50 |
|  |  | H3 | 216.65 | 5197.73 | 2211.74 | 1225.45 |
|  |  |  |  |  |  |  |
| Growth habit | 2024 | H1 | 2.55 | 8.02 | 4.77 | 0.99 |
| Plant height, cm |  | H1 | 16.84 | 48.49 | 29.49 | 5.41 |
|  |  | H2 | 26.92 | 73.99 | 42.93 | 7.54 |
|  |  | H3 | 37.11 | 92.05 | 62.38 | 8.33 |
| Ground cover |  | H1 | 3.46 | 8.65 | 6.51 | 0.66 |
|  |  | H2 | 2.92 | 7.36 | 5.15 | 0.88 |
|  |  | H3 | 1.87 | 8.03 | 5.01 | 1.22 |
| DMY, kg/ha |  | H1 | 302.43 | 1840.99 | 829.95 | 340.55 |
|  |  | H2 | 765.33 | 4185.70 | 2059.03 | 683.56 |
|  |  | H3 | 852.65 | 5515.38 | 3088.75 | 838.33 |
|  |  |  |  |  |  |  |
| Growth habit | 2025 | H1 | 1.85 | 8.54 | 5.51 | 1.13 |
| Plant height, cm |  | H1 | 28.65 | 80.72 | 53.62 | 7.80 |
|  |  | H2 | 25.10 | 52.20 | 37.34 | 4.50 |
|  |  | H3 | 36.46 | 70.02 | 50.40 | 5.48 |
| Ground cover |  | H1 | 5.44 | 9.16 | 7.26 | 0.70 |
|  |  | H2 | 3.83 | 9.12 | 6.38 | 0.77 |
|  |  | H3 | 2.51 | 8.34 | 5.82 | 0.95 |
| DMY, kg/ha |  | H1 | 407.40 | 3955.49 | 2121.70 | 590.99 |
|  |  | H2 | 580.81 | 2567.67 | 1620.61 | 343.23 |
|  |  | H3 | 649.65 | 4228.92 | 2401.50 | 605.29 |

H1, H2, and H3 – biomass harvests 1 – 3, SD – standard deviation, H^2^ – broad sense heritability,

DMY – dry matter yield.

| Trait | Year | Harvest | H^2^ |
| --- | --- | --- | --- |
| Growth habit | 2023 | H1 | 0.77 |
| Plant height, cm |  | H2 | 0.39 |
|  |  | H3 | 0.49 |
| DMY, kg/ha |  | H1 | 0.30 |
|  |  | H2 | 0.37 |
|  |  | H3 | 0.39 |
|  |  |  |  |
| Growth habit | 2024 | H1 | 0.81 |
| Plant height, cm |  | H1 | 0.73 |
|  |  | H2 | 0.77 |
|  |  | H3 | 0.38 |
| Ground cover |  | H1 | 0.24 |
|  |  | H2 | 0.53 |
|  |  | H3 | 0.60 |
| DMY, kg/ha |  | H1 | 0.44 |
|  |  | H2 | 0.47 |
|  |  | H3 | 0.42 |
|  |  |  |  |
| Growth habit | 2025 | H1 | 0.73 |
| Plant height, cm |  | H1 | 0.71 |
|  |  | H2 | 0.27 |
|  |  | H3 | 0.34 |
| Ground cover |  | H1 | 0.38 |
|  |  | H2 | 0.58 |
|  |  | H3 | 0.46 |
| DMY, kg/ha |  | H1 | 0.31 |
|  |  | H2 | 0.32 |
|  |  | H3 | 0.32 |
|  |  |  |  |
| Growth habit | Across environments |  | 0.55 |
| Plant height, cm |  |  | 0.43 |
| Ground cover |  |  | 0.63 |
| DMY, kg/ha |  |  | 0.16 |

Supplementary Table S4. Heritability of biomass-related traits for each harvest and across environments.

H1, H2, and H3 – biomass harvests 1 – 3, H^2^ – broad sense heritability,

DMY – dry matter yield.

Supplementary Table S5: Predictive abilities of dry matter yield using varying population sizes in different harvest periods and years.

| Year | Marker platform | Training population sizes | H1 | H2 | H3 |
| --- | --- | --- | --- | --- | --- |
| 2023 | 3K | 50 | 0.160 | 0.034 | 0.191 |
|  |  | 100 | 0.174 | 0.074 | 0.220 |
|  |  | 150 | 0.191 | 0.081 | 0.247 |
|  |  | 200 | 0.193 | 0.096 | 0.272 |
|  |  | 250 | 0.221 | 0.114 | 0.275 |
|  |  | 300 | 0.233 | 0.112 | 0.277 |
|  |  | 350 | 0.238 | 0.082 | 0.281 |
|  |  |  |  |  |  |
|  | GBS | 50 | 0.147 | 0.033 | 0.192 |
|  |  | 100 | 0.156 | 0.070 | 0.243 |
|  |  | 150 | 0.179 | 0.088 | 0.275 |
|  |  | 200 | 0.179 | 0.105 | 0.303 |
|  |  | 250 | 0.204 | 0.118 | 0.310 |
|  |  | 300 | 0.222 | 0.111 | 0.295 |
|  |  | 350 | 0.210 | 0.088 | 0.297 |
|  |  |  |  |  |  |
| 2024 | 3K | 50 | 0.355 | 0.193 | 0.108 |
|  |  | 100 | 0.401 | 0.248 | 0.167 |
|  |  | 150 | 0.438 | 0.259 | 0.180 |
|  |  | 200 | 0.450 | 0.275 | 0.204 |
|  |  | 250 | 0.446 | 0.278 | 0.211 |
|  |  | 300 | 0.456 | 0.287 | 0.205 |
|  |  | 350 | 0.461 | 0.302 | 0.199 |
|  |  |  |  |  |  |
|  | GBS | 50 | 0.312 | 0.200 | 0.110 |
|  |  | 100 | 0.374 | 0.270 | 0.162 |
|  |  | 150 | 0.414 | 0.295 | 0.192 |
|  |  | 200 | 0.436 | 0.307 | 0.225 |
|  |  | 250 | 0.429 | 0.321 | 0.236 |
|  |  | 300 | 0.448 | 0.330 | 0.233 |
|  |  | 350 | 0.447 | 0.343 | 0.236 |
|  |  |  |  |  |  |
| 2025 | 3K | 50 | 0.083 | 0.048 | 0.075 |
|  |  | 100 | 0.114 | 0.056 | 0.097 |
|  |  | 150 | 0.125 | 0.052 | 0.131 |
|  |  | 200 | 0.139 | 0.071 | 0.142 |
|  |  | 250 | 0.164 | 0.081 | 0.147 |
|  |  | 300 | 0.175 | 0.067 | 0.149 |
|  |  | 350 | 0.196 | 0.045 | 0.181 |
|  |  |  |  |  |  |
|  | GBS | 50 | 0.112 | 0.036 | 0.064 |
|  |  | 100 | 0.150 | 0.033 | 0.092 |
|  |  | 150 | 0.168 | 0.027 | 0.116 |
|  |  | 200 | 0.185 | 0.044 | 0.132 |
|  |  | 250 | 0.194 | 0.062 | 0.138 |
|  |  | 300 | 0.206 | 0.049 | 0.147 |
|  |  | 350 | 0.234 | 0.035 | 0.172 |

H1, H2 and H3 – Biomass harvests 1 – 3, 3K – 3K SNP array-based marker platform, GBS – genotyping by sequencing SNP marker platform.

Supplementary Table S6: Effects of varying marker numbers on prediction accuracies of dry matter yield comparing GBS and 3k marker platforms.

| Harvest 1 2024 | | | | | | | |
| --- | --- | --- | --- | --- | --- | --- | --- |
| Marker Number | GBS | | |  | 3K |  |  |
|  | Min | Max | Mean |  | Min | Max | Mean |
| 20 | 0.099 | 0.556 | 0.335 |  | 0.111 | 0.458 | 0.276 |
| 100 | 0.236 | 0.479 | 0.360 |  | 0.230 | 0.530 | 0.363 |
| 200 | 0.296 | 0.532 | 0.419 |  | 0.308 | 0.641 | 0.428 |
| 500 | 0.318 | 0.545 | 0.439 |  | 0.308 | 0.633 | 0.454 |
| 800 | 0.308 | 0.572 | 0.437 |  | 0.286 | 0.595 | 0.441 |
| 1200 | 0.282 | 0.571 | 0.438 |  | 0.314 | 0.613 | 0.457 |
| 1801 | 0.291 | 0.580 | 0.448 |  | 0.318 | 0.616 | 0.466 |
| 3000 | 0.291 | 0.573 | 0.439 |  | - | - | - |
| 5000 | 0.287 | 0.581 | 0.435 |  | - | - | - |
| 8000 | 0.292 | 0.607 | 0.446 |  | - | - | - |
| 12415 | 0.286 | 0.589 | 0.434 |  | - | - | - |
|  |  |  |  |  |  |  |  |
| Harvest 2 2023 | | | | | | | |
| Marker Number | GBS | | |  | 3K | | |
|  | Min | Max | Mean |  | Min | Max | Mean |
| 20 | -0.140 | 0.357 | 0.114 |  | -0.097 | 0.353 | 0.105 |
| 100 | -0.093 | 0.306 | 0.086 |  | -0.084 | 0.347 | 0.125 |
| 200 | -0.089 | 0.324 | 0.097 |  | -0.051 | 0.400 | 0.138 |
| 500 | -0.109 | 0.428 | 0.112 |  | -0.043 | 0.394 | 0.117 |
| 800 | -0.096 | 0.435 | 0.123 |  | -0.013 | 0.407 | 0.122 |
| 1200 | -0.108 | 0.411 | 0.114 |  | -0.047 | 0.411 | 0.114 |
| 1801 | -0.086 | 0.382 | 0.114 |  | -0.040 | 0.413 | 0.124 |
| 3000 | -0.083 | 0.388 | 0.099 |  | - | - | - |
| 5000 | -0.080 | 0.405 | 0.110 |  | - | - | - |
| 8000 | -0.080 | 0.406 | 0.122 |  | - | - | - |
| 12415 | -0.072 | 0.419 | 0.112 |  | - | - | - |

2024-harvest 1 – environment with the highest predictive ability and 2023-harvest 2 – environment with the lowest predictive ability for dry matter yield.

Supplementary table S7. Predictive abilities of dry matter yield across harvests and years based on linear and machine learning models in cross validations.

| Year | SNP assay | Harvest |  | Methods |  |  |
| --- | --- | --- | --- | --- | --- | --- |
|  |  |  | GBLUP | BB | RF | GB |
| 2023 | 3K | H1 | 0.252 ± 0.015 | 0.252 ± 0.015 | 0.160 ± 0.016 | 0.097 ± 0.015 |
|  |  | H2 | 0.071 ± 0.018 | 0.0489 ± 0.016 | 0.014 ± 0.015 | 0.004 ± 0.017 |
|  |  | H3 | 0.229 ± 0.015 | 0.226 ± 0.014 | 0.241 ± 0.015 | 0.179 ± 0.016 |
|  |  |  |  |  |  |  |
|  | GBS | H1 | 0.225 ± 0.014 | 0.219 ± 0.015 | 0.130 ± 0.016 | 0.060 ± 0.014 |
|  |  | H2 | 0.058 ± 0.018 | 0.002 ± 0.017 | 0.012 ± 0.017 | 0.0088 ± 0.018 |
|  |  | H3 | 0.277 ± 0.015 | 0.274 ± 0.015 | 0.310 ± 0.013 | 0.201 ± 0.013 |
|  |  |  |  |  |  |  |
| 2024 | 3K | H1 | 0.480 ± 0.011 | 0.490 ± 0.011 | 0.464 ± 0.011 | 0.364 ± 0.012 |
|  |  | H2 | 0.281 ± 0.013 | 0.276 ± 0.013 | 0.217 ± 0.014 | 0.144 ± 0.014 |
|  |  | H3 | 0.234 ± 0.013 | 0.232 ± 0.013 | 0.178 ± 0.014 | 0.133 ± 0.011 |
|  |  |  |  |  |  |  |
|  | GBS | H1 | 0.461 ± 0.010 | 0.462 ± 0.010 | 0.490 ± 0.010 | 0.402 ± 0.012 |
|  |  | H2 | 0.330 ± 0.011 | 0.324 ± 0.012 | 0.332 ± 0.013 | 0.256 ± 0.013 |
|  |  | H3 | 0.270 ± 0.011 | 0.270 ± 0.011 | 0.256 ± 0.013 | 0.201 ± 0.012 |
|  |  |  |  |  |  |  |
| 2025 | 3K | H1 | 0.155 ± 0.015 | 0.145 ± 0.015 | 0.119 ± 0.015 | 0.103 ± 0.014 |
|  |  | H2 | 0.073 ± 0.016 | 0.066 ± 0.016 | 0.070 ± 0.016 | 0.050 ± 0.015 |
|  |  | H3 | 0.159 ± 0.015 | 0.160 ± 0.016 | 0.201 ± 0.017 | 0.171 ± 0.016 |
|  |  |  |  |  |  |  |
|  | GBS | H1 | 0.185 ± 0.016 | 0.165 ± 0.016 | 0.159 ± 0.014 | 0.104 ± 0.015 |
|  |  | H2 | 0.070 ± 0.014 | 0.066 ± 0.014 | 0.080 ± 0.013 | 0.046 ± 0.014 |
|  |  | H3 | 0.155 ± 0.014 | 0.171 ± 0.016 | 0.222 ± 0.016 | 0.174 ± 0.017 |

GBLUP – genomic best linear unbiased prediction, RF – random forest regression, XGBoost – extreme	gradient boosting regression, 3K – 3K SNP array-based marker platform, GBS – genotyping by sequencing	SNP marker platform.

| Model | Harvest | 2023 | | 2024 | | 2025 | |
| --- | --- | --- | --- | --- | --- | --- | --- |
|  |  | 3k | GBS | 3k | GBS | 3k | GBS |
| G | H1 | 0.232 ± 0.010 | 0.221 ± 0.011 | 0.481 ± 0.008 | 0.456 ± 0.007 | 0.162 ± 0.009 | 0.191 ± 0.009 |
| G + GH |  | 0.388 ± 0.010 | 0.388 ± 0.011 | 0.661 ± 0.006 | 0.655 ± 0.007 | 0.493 ± 0.008 | 0.507 ± 0.008 |
| G + PH |  | - | - | 0.682 ± 0.005 | 0.676 ± 0.006 | 0.627 ± 0.006 | 0.634 ± 0.006 |
| G + GC |  | - | - | 0.583 ± 0.007 | 0.564 ± 0.007 | 0.306 ± 0.007 | 0.316 ± 0.008 |
| G + GH +PH |  | - | - | 0.697 ± 0.005 | 0.692 ± 0.006 | 0.632 ± 0.006 | 0.640 ± 0.006 |
| G + GC +PH |  | - | - | 0.724 ± 0.005 | 0.716 ± 0.005 | 0.657 ± 0.006 | 0.663 ± 0.005 |
| G + GC +GH |  | - | - | 0.730 ± 0.005 | 0.721 ± 0.005 | 0.639 ± 0.006 | 0.647 ± 0.006 |
| G + GH +PH + GC |  | - | - | 0.746 ± 0.005 | 0.739 ± 0.005 | 0.686 ± 0.005 | 0.693 ± 0.005 |
|  |  |  |  |  |  |  |  |
| G | H2 | 0.120 ± 0.009 | 0.118 ± 0.010 | 0.281 ± 0.009 | 0.326 ± 0.008 | 0.074 ± 0.010 | 0.054 ± 0.010 |
| G + GH |  | 0.217 ± 0.009 | 0.199 ± 0.010 | 0.471 ± 0.007 | 0.484 ± 0.006 | 0.202 ± 0.012 | 0.198 ± 0.012 |
| G + PH |  | 0.509 ± 0.008 | 0.499 ± 0.008 | 0.724 ± 0.006 | 0.726 ± 0.006 | 0.451 ± 0.009 | 0.452 ± 0.009 |
| G + GC |  | - | - | 0.305 ± 0.008 | 0.357 ± 0.008 | 0.294 ± 0.009 | 0.301 ± 0.010 |
| G + GH + PH |  | 0.505 ± 0.008 | 0.495 ± 0.008 | 0.724 ± 0.006 | 0.725 ± 0.006 | 0.497 ± 0.009 | 0.497 ± 0.009 |
| G + GC + PH |  | - | - | 0.746 ± 0.006 | 0.747 ± 0.006 | 0.550 ± 0.008 | 0.554 ± 0.008 |
| G + GC + GH |  | - | - | 0.595 ± 0.006 | 0.603 ± 0.005 | 0.294 ± 0.010 | 0.301 ± 0.010 |
| G + PH+ GH + GC |  | - | - | 0.763 ± 0.005 | 0.762 ± 0.005 | 0.549 ± 0.008 | 0.553 ± 0.008 |
|  |  |  |  |  |  |  |  |
| G | H3 | 0.289 ± 0.009 | 0.329 ± 0.009 | 0.237 ± 0.009 | 0.259 ± 0.010 | 0.164 ± 0.010 | 0.160 ± 0.010 |
| G + GH |  | 0.273 ± 0.007 | 0.314 ± 0.007 | 0.331 ± 0.009 | 0.348 ± 0.009 | 0.281 ± 0.009 | 0.295 ± 0.007 |
| G + PH |  | 0.516 ± 0.007 | 0.520 ± 0.007 | 0.435 ± 0.009 | 0.448 ± 0.009 | 0.570 ± 0.007 | 0.570 ± 0.007 |
| G + GC |  | - | - | 0.734 ± 0.005 | 0.735 ± 0.005 | 0.594 ± 0.007 | 0.592 ± 0.007 |
| G + GH + PH |  | 0.516 ± 0.007 | 0.518 ± 0.007 | 0.479 ± 0.008 | 0.491 ± 0.008 | 0.597 ± 0.006 | 0.599 ± 0.006 |
| G + GC + PH |  | - | - | 0.763 ± 0.004 | 0.764 ± 0.004 | 0.678 ± 0.006 | 0.675 ± 0.006 |
| G + GC + GH |  | - | - | 0.732 ± 0.005 | 0.733 ± 0.005 | 0.596 ± 0.007 | 0.595 ±0.007 |
| G + PH+ GH + GC |  | - | - | 0.761 ± 0.004 | 0.762 ±0.004 | 0.682 ± 0.006 | 0.679 ± 0.006 |

Supplementary Table S8. Mean predictive abilities of dry matter yield for univariate and multivariate GBLUP prediction models with different combinations of secondary traits.

H1, H2, and H3 – biomass harvests 1 – 3, GH – growth habit, PH – plant height, GC – ground cover, G – genomic data, 3K – 3K SNP array-based marker platform, GBS – genotyping by sequencing SNP marker platform.

Supplementary Table S9. Mean predictive abilities of dry matter yield for univariate and multivariate random forest prediction models with different combinations of secondary traits.

| Model | Harvest | 2023 | | 2024 | | 2025 | |
| --- | --- | --- | --- | --- | --- | --- | --- |
|  |  | 3k | GBS | 3k | GBS | 3k | GBS |
| G | H1 | 0.158 ± 0.009 | 0.131 ± 0.010 | 0.458 ± 0.008 | 0.496 ± 0.007 | 0.108 ± 0.008 | 0.160 ± 0.008 |
| G + GH |  | 0.347 ± 0.009 | 0.331 ± 0.009 | 0.617 ± 0.006 | 0.609 ± 0.007 | 0.384 ± 0.007 | 0.401 ± 0.007 |
| G + PH |  | - | - | 0.6512 ± 0.005 | 0.645 ± 0.006 | 0.564 ± 0.006 | 0.549 ± 0.006 |
| G + GC |  | - | - | 0.575 ± 0.006 | 0.561 ± 0.007 | 0.212 ± 0.007 | 0.235 ± 0.007 |
| G + GH +PH |  | - | - | 0.673 ± 0.005 | 0.661 ± 0.006 | 0.590 ± 0.006 | 0.579 ± 0.006 |
| G + GC +PH |  | - | - | 0.702 ± 0.004 | 0.687 ± 0.005 | 0.596 ± 0.006 | 0.574 ± 0.006 |
| G + GC +GH |  | - | - | 0.693 ± 0.005 | 0.670 ± 0.006 | 0.509 ± 0.006 | 0.490 ± 0.007 |
| G + GH +PH + GC |  | - | - | 0.722 ± 0.005 | 0.701 ± 0.005 | 0.630 ± 0.006 | 0.609 ± 0.006 |
|  |  |  |  |  |  |  |  |
| G | H2 | 0.050 ± 0.009 | 0.042 ± 0.009 | 0.226 ± 0.010 | 0.329 ± 0.008 | 0.080 ± 0.009 | 0.085 ± 0.009 |
| G + GH |  | 0.074 ± 0.009 | 0.047 ± 0.009 | 0.390 ± 0.008 | 0.437 ± 0.007 | 0.135 ± 0.009 | 0.121 ± 0.009 |
| G + PH |  | 0.400 ± 0.010 | 0.375 ± 0.009 | 0.669 ± 0.005 | 0.681 ± 0.005 | 0.393 ± 0.008 | 0.393 ± 0.008 |
| G + GC |  | - | - | 0.259 ± 0.009 | 0.339 ± 0.008 | 0.170 ± 0.009 | 0.200 ± 0.009 |
| G + GH + PH |  | 0.405 ± 0.009 | 0.378 ± 0.009 | 0.683 ± 0.005 | 0.691 ± 0.005 | 0.418 ± 0.008 | 0.407 ± 0.008 |
| G + GC + PH |  | - | - | 0.687 ± 0.005 | 0.690 ± 0.005 | 0.460 ± 0.008 | 0.462 ± 0.008 |
| G + GC + GH |  | - | - | 0.467 ± 0.007 | 0.478 ± 0.007 | 0.192 ± 0.009 | 0.214 ± 0.009 |
| G + PH+ GH + GC |  | - | - | 0.708 ± 0.005 | 0.704 ± 0.004 | 0.466 ± 0.008 | 0.466 ± 0.007 |
|  |  |  |  |  |  |  |  |
| G | H3 | 0.315 ± 0.007 | 0.336 ± 0.008 | 0.175 ± 0.009 | 0.254 ± 0.009 | 0.205 ± 0.009 | 0.229 ± 0.009 |
| G + GH |  | 0.315 ± 0.007 | 0.336 ± 0.008 | 0.242 ± 0.008 | 0.290 ± 0.008 | 0.248 ± 0.009 | 0.260 ± 0.009 |
| G + PH |  | 0.506 ± 0.007 | 0.508 ± 0.007 | 0.343 ± 0.008 | 0.368 ± 0.008 | 0.532 ± 0.007 | 0.534 ± 0.007 |
| G + GC |  | - | - | 0.676 ± 0.005 | 0.686 ± 0.005 | 0.565 ± 0.007 | 0.553 ± 0.007 |
| G + GH + PH |  | 0.508 ± 0.007 | 0.508 ± 0.007 | 0.380 ± 0.008 | 0.391 ± 0.008 | 0.560 ± 0.006 | 0.549 ± 0.006 |
| G + GC + PH |  | - | - | 0.725 ± 0.005 | 0.723 ± 0.005 | 0.651 ± 0.006 | 0.637 ± 0.006 |
| G + GC + GH |  | - | - | 0.679 ± 0.005 | 0.687 ± 0.005 | 0.571 ± 0.007 | 0.558 ± 0.007 |
| G + PH+ GH + GC |  | - | - | 0.724 ± 0.005 | 0.722 ± 0.005 | 0.658 ± 0.006 | 0.643 ± 0.006 |

H1, H2, and H3 – biomass harvests 1 – 3, GH – growth habit, PH – plant height, GC – ground cover, G – genomic data only, 3K – 3K SNP array-based marker platform, GBS – genotyping by sequencing SNP marker platform.

Supplementary Table S10. Forward predictive abilities of dry matter yield in different harvest periods within each year.

| Year | SNP assay | Training | Testing | PA |
| --- | --- | --- | --- | --- |
| 2023 | 3K | H1 | H2 | 0.122 |
|  |  | H1 | H3 | 0.094 |
|  |  | H2 | H3 | 0.112 |
|  |  | H1_H2 | H3 | 0.118 |
|  |  |  |  |  |
|  | GBS | H1 | H2 | 0.141 |
|  |  | H1 | H3 | 0.012 |
|  |  | H2 | H3 | 0.085 |
|  |  | H1_H2 | H3 | 0.077 |
|  |  |  |  |  |
| 2024 | 3K | H1 | H2 | 0.380 |
|  |  | H1 | H3 | 0.207 |
|  |  | H2 | H3 | 0.169 |
|  |  | H1_H2 | H3 | 0.187 |
|  |  |  |  |  |
|  | GBS | H1 | H2 | 0.415 |
|  |  | H1 | H3 | 0.190 |
|  |  | H2 | H3 | 0.154 |
|  |  | H1_H2 | H3 | 0.167 |
|  |  |  |  |  |
| 2025 | 3K | H1 | H2 | 0.040 |
|  |  | H1 | H3 | 0.023 |
|  |  | H2 | H3 | 0.049 |
|  |  | H1_H2 | H3 | 0.021 |
|  |  |  |  |  |
|  | GBS | H1 | H2 | 0.049 |
|  |  | H1 | H3 | 0.022 |
|  |  | H2 | H3 | 0.048 |
|  |  | H1_H2 | H3 | 0.020 |

H1, H2, and H3 – biomass harvests 1 – 3, 3K – 3K SNP array-based marker platform, GBS – genotyping by sequencing SNP marker platform, PA – predictive abilities.
